# Post-mortem evidence of pathogenic angiogenesis and abnormal vascular function in early Alzheimer’s disease

**DOI:** 10.1093/brain/awaf394

**Published:** 2025-10-22

**Authors:** Daniel J Asby, Robert A Fisher, Johanna Jackson, Seth Love, Paul M Matthews, J Scott Miners

**Affiliations:** Translational Health Sciences, Cerebrovascular & Dementia Research Group, Bristol Medical School, University of Bristol, Bristol, BS10 5NB, UK; Translational Health Sciences, Cerebrovascular & Dementia Research Group, Bristol Medical School, University of Bristol, Bristol, BS10 5NB, UK; Department of Brain Sciences, UK Dementia Research Institute at Imperial College London, London, W12 0BZ, UK; Department of Brain Sciences, Faculty of Medicine, Imperial College London, London, W12 0NN, UK; Translational Health Sciences, Cerebrovascular & Dementia Research Group, Bristol Medical School, University of Bristol, Bristol, BS10 5NB, UK; Department of Brain Sciences, UK Dementia Research Institute at Imperial College London, London, W12 0BZ, UK; Department of Brain Sciences, Faculty of Medicine, Imperial College London, London, W12 0NN, UK; Harwell Science and Innovation Campus, Rosalind Franklin Institute, Didcot, OX11 0QX, UK; Translational Health Sciences, Cerebrovascular & Dementia Research Group, Bristol Medical School, University of Bristol, Bristol, BS10 5NB, UK

**Keywords:** cerebral hypoperfusion, blood–brain barrier (BBB), angiogenesis, pericyte, vascular endothelial growth factor-A, endothelin-1

## Abstract

Cerebral hypoperfusion and blood–brain barrier (BBB) leakiness are pathological features of Alzheimer’s disease (AD). To understand their relationship to the distribution and progression of Alzheimer’s disease neuropathologic change (ADNC), we analysed associations between biochemical markers and mediators of cerebral hypoperfusion and BBB leakiness, and amyloid-β (Aβ) and hyperphosphorylated tau, in multiple brain regions at different Braak tangle stages (BS).

We studied the frontal, temporal, parietal, entorhinal, calcarine and cingulate cortex, putamen and trigonal white matter from eight controls with low pathology (BS0–II), 17 brains with early-stage/intermediate AD pathology (BSIII–IV) and 11 late-stage AD cases (BSV–VI), from the South West Dementia Brain Bank and the London Neurodegenerative Diseases Brain Bank. We excluded cases with widespread moderate-severe arteriolosclerosis, macroscopic infarcts or foci of haemorrhage, Lewy body pathology or other neurodegenerative pathology. ELISAs were used to measure the myelin-associated glycoprotein:proteolipid protein-1 ratio (MAG:PLP1), an index of ante-mortem cerebral perfusion, and fibrinogen levels, to assess BBB leakiness. Also by ELISA, we measured vascular endothelial growth factor-A (VEGF-A), upregulated in cerebral ischaemia; endothelin-1 (EDN1), a mediator of vasoconstriction; CD31, an endothelial marker; platelet-derived growth factor-β (PDGFRβ), a pericyte marker; and Aβ_1-40_, Aβ_1–42_ and ptau-231 concentrations. In the temporal cortex from a subset of cases, 55 angiogenesis-related proteins were assayed using a multiplex profiler assay.

MAG:PLP1 was lower in BSIII–IV than BS0–II in all brain regions. VEGF-A, EDN1 and CD31 concentrations were highest in BSIII–IV in most regions and correlated inversely with MAG:PLP1. EDN1 level correlated strongly with Aβ_1–42_ concentration in low-pathology controls. Angiogenesis-related protein assays showed elevated levels of endoglin (CD105), a marker of neoangiogenesis, in BSIII–IV, coinciding with altered expression of several angiogenic mediators. The PDGFRβ:CD31 ratio, a marker of pericyte content adjusted for vessel density, was lower in BSIII–IV than BS0–II and correlated positively with MAG:PLP1 and inversely with Aβ_1–42_. BBB leakiness, evidenced by elevated fibrinogen in brain tissue homogenates, was highest in BSV–VI in most brain regions and correlated with VEGF-A, CD31 and ptau-231 concentrations.

The present data provide evidence of widespread cerebral hypoperfusion associated with pathogenic angiogenesis and vascular remodelling in AD. The study highlights a complex and dynamic temporal relationship, beginning in early-stage AD, between mediators of cerebrovascular dysfunction and the regional spread of Aβ and tau pathology. The study also identifies several therapeutic targets, including EDN1 and VEGF-A signalling, with the potential to limit cerebrovascular damage in early AD.

## Teaser

By analysing post-mortem human brain tissue, Asby *et al.* found that reduced blood supply in Alzheimer's disease promotes the growth of new but leaky blood vessels. These vascular changes are widespread even in the early stages of the disease and worsen as amyloid and tau pathology accumulate.

**See Kalaria (https://doi.org/10.1093/brain/awag076) for a scientific commentary on this article.**

## Introduction

Cerebrovascular pathology underpins vascular dementia but is also a prominent feature in Alzheimer’s disease (AD). Cerebral amyloid angiopathy (CAA),^1,2^ non-amyloid cerebral small vessel disease (cSVD) and ischaemic white matter changes^3-5^ often co-exist in AD. White matter damage and cerebral ischaemia as a result of cSVD can be detected up to 30 years prior to the predicted onset of cognitive decline in AD.^6^ Reduced blood flow within the precuneus is detectable up to 10–15 years before the predicted onset of clinical symptoms in familial AD and the subsequent spread of cerebral hypoperfusion approximately mirrors the regional deposition of amyloid-Aβ (Aβ).^7^ Cerebral ischaemia is linked to the overproduction of Aβ (reviewed in Love and Miners^8^) and hyperphosphorylation of tau (reviewed in Pluta *et al*.^9^). Oligomeric Aβ peptides mediate vasoconstriction through oxidative stress-induced, endothelin-1 (EDN1)-mediated pericyte constriction^7,10^ and pathogenic tau accumulates around cerebrocortical blood vessels^11,12^ and disrupts calcium signalling and endothelium-dependent vasodilation.^13^ Both Aβ and tau peptides induce blood–brain barrier (BBB) leakiness *in vitro* and *in vivo*,^14-16^ and BBB leakiness correlates with Aβ and tau load in clinical studies.^17^ BBB breakdown contributes to cognitive decline and is evident within the hippocampus in the very early stages of AD.^18,19^ Together, these data indicate a clinically relevant, bi-directional relationship between cerebrovascular dysfunction and Aβ and tau pathology that begins in early AD.

Single-cell molecular atlases of the cerebral vasculature have provided further evidence for a pathogenic role of cerebrovascular dysfunction in AD. Pericyte and endothelial cells express injury-related gene signatures in AD, and differential gene and pathway analyses confirm that pathways associated with angiogenesis, vasoconstriction and BBB leakiness play a key role in AD pathogenesis.^20,21^ These studies have also revealed that AD-risk genes are enriched within endothelial cells, as well as microglia. A single nuclei RNA (snRNA) transcriptomic analysis of six brain regions identified heterogeneity of the vascular transcriptome across brain regions, with lower proportions of pericytes and endothelial cells in the entorhinal cortex and hippocampus and regional differences in gene expression in pathways associated with BBB leakiness.^22^

We previously investigated the pathophysiology of cerebral hypoperfusion and BBB leakiness in dementia, through the analysis of human post-mortem brain tissue by measurement of biochemical markers that provide information on vascular function. Since myelin-associated glycoprotein (MAG) is more susceptible than proteolipid protein-1 (PLP1) to tissue hypoxia, and the turnover of both MAG and PLP1 myelin proteins is slow (half-lives of ∼3 months), a reduction in brain MAG:PLP1 is a marker of reduced ante-mortem tissue perfusion over several months prior to death (reviewed in Love and Miners^8^). We previously showed MAG:PLP1 to be reduced in vascular dementia in relation to cSVD severity.^23^ We also observed a significant reduction in MAG:PLP1 in AD in several independent cohorts,^24,25^ the precuneus being affected in early-intermediate [Braak tangle stage (BS)III–IV] AD.^24^ Across these studies, reduced MAG:PLP1 correlated inversely with vascular endothelial growth factor-A (VEGF-A),^26^ a HIF-1α-dependent protein that is rapidly induced in response to reduced tissue oxygenation, and is a central mediator of angiogenesis and BBB permeability. We also reported that lower MAG:PLP1 correlated inversely with the concentration of the potent vasoconstrictor peptide, EDN1, in AD^24^ and higher EDN1 levels were associated with elevated Aβ_1–42_.^27^ Within the precuneus, an area subject to cerebral hypoperfusion early in AD, lower MAG:PLP1 and higher VEGF-A correlated with loss of the pericyte protein, platelet-derived growth factor β (PDGFRβ), and with elevated fibrinogen, reflecting BBB leakiness.^25^ PDGFRβ and fibrinogen levels also correlated with Aβ plaque load, and Aβ_1–42_ and Aβ_1–40_ concentrations, respectively. In a mixed-dementia cohort, EDN1 and VEGF-A concentrations were inversely related to MAG:PLP1 and parenchymal Aβ load, and correlated positively with fibrinogen.^28^

In the present study, we have investigated the relationships between markers of cerebrovascular function (MAG:PLP1, VEGF-A, EDN1, PDGFRβ:CD31, fibrinogen) and disease pathology (Aβ_1–40_, Aβ_1–42_ and ptau-231) in eight cortical and subcortical regions in brains stratified by Braak tangle stage (BS) into low-pathology controls (BS0–II), early-stage/intermediate pathology (BSIII–IV) and late-stage AD (BSV–VI). Our findings provide further evidence for widespread cerebral hypoperfusion in early-stage AD, associated with elevated EDN1, which is related to higher Aβ_1–42_. A lower MAG:PLP1 ratio was associated with higher VEGF-A and CD31 concentrations in BSIII–IV. Lower concentrations of the pericyte marker PDGFRβ and elevated levels of the neoangiogenesis marker, endoglin, suggesting vascular remodelling in early-stage AD. Late-stage AD was associated with dysregulation of mediators of angiogenesis and elevated fibrinogen levels, indicative of BBB leakiness, associated with ptau-231. Our study provides novel insights into the pathophysiological pathways that underpin widespread cerebrovascular dysfunction in AD, and their close relationships to Aβ and tau.

## Materials and methods

### Study cohort

We studied eight low-pathology (BS0–II) controls, 17 early-stage/intermediate pathology (BSIII–IV) and 11 late-stage pathology (BSV–VI) brains in eight regions: frontal cortex [Brodmann areas 9 and 46 (BA9/BA46)], cingulate cortex (BA24/32), entorhinal cortex (BA28/BA34), putamen, temporal cortex (BA21/22), parietal (BA40) and calcarine (BA17) cortex, and trigonal white matter. AD cases were diagnosed according to National Institute on Aging and Alzheimer's Association guidelines^29^ after exclusion of Lewy body pathology or other neurodegenerative pathology apart from early-stage limbic-predominant age-related TDP-43 encapthalopathy. Cases of widespread moderate-severe arteriolosclerosis, macroscopic infarcts or foci of haemorrhage, were excluded. Age-at-death and post-mortem delay were matched as closely as possible across the three groups. The demographic and neuropathological features of the study cohort are summarized in Table 1 and shown in full in Supplementary Table 1. A list of unique UK brain bank identifiers for all cases in this study are provided in Supplementary Table 16.

**Table 1:** Study demographic and neuropathology

ID	Age, years	Sex	PM, h	CERAD	CERAD np	BS	Thal
**Low-pathology controls**							
1	93	Male	34.5	No AD	None	2	1
2	75	Male	46.5	No AD	None	2	4
3	92	Female	71	No AD	None	2	4
4	87	Female	29.5	Possible	Sparse	2	4
5	81	Female	42	No AD	None	0	0
6	80	Male	58	No AD	None	1	0
7	69	Female	48	No AD	Sparse	0	1
8	95	Male	18	No AD	Sparse	2	5
*Mean*	*84.0*	*4 Male*	*43.4*	*–*	*–*	*1.4*	*2.4*
*SD*	*9.3*	*4 Female*	*16.6*	*–*	*–*	*0.9*	*2.1*
**Intermediate pathology AD**							
9	77	Male	41	Possible	Sparse	3	4
10	97	Female	86	Probable	Moderate	4	4
11	91	Female	74	Probable	Frequent	4	4
12	95	Female	88	Possible	Moderate	4	4
13	79	Male	76	Probable	Moderate	3	4
14	94	Female	16	Possible	Sparse	3	3
15	86	Male	35	Possible	Frequent	4	4
16	97	Female	15	Possible	Sparse	3	4
17	90	Male	31	Possible	Sparse	3	1
18	73	Male	22	Probable	Sparse	3	4
19	86	Male	56	Probable	Moderate	4	3
20	92	Male	47	Probable	Moderate	4	5
21	75	Male	105	Possible	Sparse	3	5
22	98	Male	49	Possible	Sparse	3	5
23	81	Male	38	Definite	Frequent	4	5
24	93	Male	37	Probable	Moderate	4	3
25	83	Female	41.75	Definite	Frequent	3	5
*Mean*	*87.8*	*11 Male*	*51.0*	*–*	*–*	*3.5*	*4.0*
*SD*	*8.4*	*6 Female*	*27.3*	*–*	*–*	*0.5*	*1.1*
**Late-stage pathology AD**							
26	72	Female	11.5	Definite	Frequent	6	5
27	86	Female	37.5	Definite	Frequent	6	5
28	64	Male	44.75	Definite	Frequent	6	5
29	90	Male	61	Definite	Frequent	5	4
30	72	Male	61	Definite	Frequent	6	5
31	75	Male	59	Definite	Frequent	5	5
32	93	Male	36	Intermed	Moderate	5	2
33	85	Female	35	Definite	Frequent	5	2
34	79	Male	20	Definite	Frequent	6	2
35	83	Female	58	Definite	Frequent	6	2
36	75	Male	36	Definite	Frequent	5	4
*Mean*	*79.5*	*7 Male*	*41.8*	*–*	*–*	*5.6*	*3.7*
*SD*	*8.8*	*4 Female*	*16.8*	*–*	*–*	*0.5*	*1.4*

Demographic and neuropathological characteristics of the study cohort. Cases were grouped according to Braak tangle stage into low-pathology controls (BS0–II) (*n* = 8), intermediate- (BSIII–IV) (*n* = 17) and late-stage (BSV–VI) pathology AD. Sex: male, female. PM = post-mortem delay (hours). Clinical diagnosis based on Consortium to Establish a Registry for Alzheimer’s Disease (CERAD): no Alzheimer’s (No AD), possible, probable and definite. CERAD Neuropathology (np) score to assess the density of neuritic plaques: none, sparse, moderate and frequent. BS = Braak tangle stage groups: 0–II, III–IV and V–VI (but shown here in Arabic numerals, for convenience). Thal = Thal phase (0–6). All cases included in the study were Braak Lewy body stage 0 (not shown). A summary of the mean and standard deviation (SD) for age-at-death (years), PM delay (hours), Braak tangle and Thal stage is shown in italics for each of the groups. Further details are provided in Supplementary Table 1.

Frozen brain tissue was obtained from the South West Dementia Brain Bank (SWDBB) (University of Bristol) and the London Neurodegenerative Diseases Brain Bank (King’s College, London). The study was approved by the SWDBB management committee (Human Tissue Authority licence number 12273) under the terms approved by the Bristol ethical research committee (18/SW/0029). Use of brain tissue from the London Neurodegenerative Disease Brain Bank was covered by ethical approval (23/WA/0124).

### Brain tissue homogenization

Frozen brain tissue (100 mg) was homogenized at 20% w/v in 1% sodium dodecyl sulfate (SDS) lysis buffer [1% w/v SDS, 0.1 M NaCl, 0.01 M Tris-HCl (pH 7.6), 1 µM phenylmethylsulfonyl fluoride and 1 µg/ml of aprotinin] using a Precellys homogenizer (2 × 15 s at 6000 rpm) with 5–10 silica beads (2.3 mm diameter). Homogenates were centrifuged at 13 000*g* for 10 min at 4°C and supernatants were carefully removed and subaliquoted prior to storage at −80°C. These samples were used for the ELISA measurements of MAG, PLP1, VEGF-A, fibrinogen, CD31 and PDGFRβ concentrations.

Frozen brain tissue (100 mg) was sequentially homogenized, initially with Nonidet P-40 (NP40), followed by extraction of a pelleted fraction with guanidine HCl to isolate soluble and insoluble plaque-associated material, respectively, as for previous studies.^23-26,28^ Levels of Aβ_1–40,_ Aβ_1–42_ and ptau-231 levels were measured in the guanidine extracts by ELISA. EDN1 levels were measured in the NP-40 soluble fractions. The NP-40 soluble fractions were also used for the angiogenesis proteome profiler assays (see below).

Total protein was measured in duplicate for all samples by use of the Coomassie Plus Assay Kit (Sigma Aldrich #23236) according to the manufacturer’s guidelines. Samples were diluted 1:20 in 0.85% NaCl and mixed with Coomassie reagent (1:30). Absorbance was measured at 450 nm in a FLUOstar® Optima plate reader (BMG Labtech). Total protein concentration was interpolated from serial dilutions of bovine serum albumin (BSA) (2000–25 μg/ml).

### MAG:PLP1 ratio, a marker of ante-mortem brain tissue hypoxic ischaemia

The MAG level was measured by direct in-house ELISA and PLP1 level was measured using a commercially available PLP1 ELISA kit (SEA417Hu, USCN), as described in previous studies.^23-26,28^ SDS brain tissue homogenates (1%) were diluted 1 in 10 in PBS for both MAG and PLP1 assays and concentrations were adjusted for total protein content before their ratio was calculated. The use of the MAG:PLP1 ratio as a marker of ante-mortem brain tissue oxygenation was reviewed previously^8^ and has been validated in multiple independent studies.^23-26,28^

### VEGF-A sandwich ELISA

We measured the VEGF-A level using a commercially available sandwich ELISA kit according to the manufacturer’s instructions (DY293B, R&D systems), as previously described.^26^ SDS brain tissue homogenates (1%) were diluted 1:10 in 1% BSA/PBS and each sample was measured in duplicate. Absorbance was measured at 450 nm in a FLUOstar® Optima plate reader (BMG Labtech) after the addition of 2N sulfuric acid. VEGF-A concentration was interpolated from serial dilutions of recombinant human VEGF-A (2000–31.25 pg/ml) and adjusted for total protein levels.

### EDN1 sandwich ELISA

The EDN1 level was measured using a commercially available ELISA kit (QET00B, R&D systems), as reported previously.^24,28^ Brain tissue homogenates were diluted 1:50 in assay diluent and 100 μl was added to the plate in duplicate. Relative luminescence was measured in a FLUOstar® Optima plate reader (BMG Labtech). The EDN1 concentration was interpolated from a standard curve generated by assaying serial dilutions of recombinant human EDN1 (250–0.343 pg/ml) and adjusted for total protein.

### CD31 sandwich ELISA

The CD31 level, a marker of endothelial content, was measured in 1% SDS homogenates by a commercially available duoset ELISA kit, according to the manufacturer’s instructions (DY806-05, R&D systems). High-binding microplates (DY990, R&D Systems) were coated with capture antibody (1:120) and incubated for 16 h overnight. Plates were then washed five times in PBS/0.05% tween-20, and blocked for 1 h using 1% BSA diluted in PBS. Following a further five washes, brain tissue homogenates were diluted 1:50 in 1% BSA/PBS and incubated for 2 h. Plates were washed again and detection antibody (1:60) was applied for 2 h. After further washes, streptavidin-horseradish peroxidase (1:40) was added for 20 min. Plates were washed for the final time, and 3,3′,5,5′-tetramethylbenzidine substrate (#34021, ThermoFisher) added for 15 min. Absorbance was measured at 450 nm in a FLUOstar® Optima plate reader (BMG Labtech) after the addition of 2N sulfuric acid. CD31 concentration was interpolated from serial dilutions of recombinant human CD31 (10 000–156.25 pg/ml) and adjusted for total protein level. All incubations were performed at room temperature. Plates underwent five successive washes between each incubation step.

### **PDGFR**β **sandwich ELISA**

The PDGFRβ level was measured by sandwich ELISA, following the manufacturer’s instructions (DYC385, R&D Systems) and as described previously.^25,28^ SDS brain tissue homogenates (1%) were diluted 1:50 in PBS and each sample was measured in duplicate. Absorbance was read at 450 nm in a FLUOstar OPTIMA® plate reader (BMG Labtech). PDGFRβ concentration was interpolated from the standard curve derived from serial dilution of recombinant PDGFRβ (16 000–250 pg/ml), and adjusted for total protein level. To adjust for potential variation in the microvessel content of different samples, we calculated the PDGFRβ:CD31 ratio.

### Fibrinogen sandwich ELISA

The fibrinogen level was measured by commercially available sandwich ELISA (Human Fibrinogen ELISA kit, Cat. No. EH3057, Wuhan Fine Biological Technology Co.), as previously described.^25,28^ SDS brain tissue homogenates (1%) were diluted in PBS (1:125). Fibrinogen concentration was interpolated from a standard curve generated by serial dilution of recombinant human fibrinogen (600–9.375 ng/ml), and adjusted for total protein level as described for the other markers.

### Aβ_1–42_ sandwich ELISA

The Aβ_1–42_ concentration was measured in the guanidine-extracted tissue samples. We used a commercially available sandwich ELISA (DAB142, R&D systems) and followed the manufacturer’s guidelines, as previously described.^25,28,30^ To fit within the standard curve, AD samples were diluted 1:2500 and control samples were diluted 1:625. Aβ_42_ concentration was interpolated from serial dilutions of recombinant human Aβ_42_ (500–7.8 pg/ml) and corrected for sample dilution. Samples were measured in duplicate and the means calculated.

### Aβ_1–40_ sandwich ELISA

The Aβ_1–40_ level was measured in guanidine-extracted tissue samples by commercially available sandwich ELISA (DAB140, R&D systems), as per the manufacturer’s guidelines. The guanidine extracts were diluted 1 in 4000 in standard diluent supplied with the kit. Aβ_1–40_ concentration was interpolated from serial dilutions of recombinant human amyloid-β_1–40_ (1000–15.625 pg/ml). Samples were measured in duplicate and the means calculated.

### ptau-231 sandwich ELISA

Phosphorylated tau-231 (ptau-231) level was measured in guanidine extracts by a commercially available sandwich ELISA kit (Cat. No. KHB8051; ThermoFisher). Tissue samples were diluted 1 in 100 in proprietary dilution buffer supplied with the kit. The concentration of ptau-231 was interpolated from a serial dilution of recombinant human ptau-231 (1000–1.562 U/ml) and corrected for sample dilution. Samples were measured in duplicate and the means calculated.

### Angiogenic proteomic profiler

Fifty-five proteins related to angiogenesis were measured using the Proteome Profiler Human Angiogenesis Array kit (Cat. No. ARY007, R&D systems), in NP40-soluble extracts of the temporal cortex. Comparisons were made between low-pathology (*n* = 3), early-stage/intermediate AD pathology (*n* = 3) and late-stage AD pathology (*n* = 4) subgroups. Brain tissue homogenates were diluted to 200 μg total protein, added to pre-loaded blots and left overnight at 4°C on a rocking platform. Blots were imaged using the Bio-Rad ChemiDoc system and densitometric analysis of images was carried out in ImageJ. The demographic and neuropathological features of this subcohort are provided in Supplementary Table 2.

### Statistical analyses

For each marker, we included carry-over samples across all plates to check for plate-to-plate variation. Biochemical analysis of vascular and pathological markers was undertaken at different time points for the Bristol and London brain bank samples. Although the patterns of BS-related changes in the concentrations of different markers were similar in both sets of samples, we noted slight but consistent variations in the absolute values recorded for some of the markers. The differences may be accounted for by our use of different batches of reagents and ELISA kits for some of the assays. This did not affect protein ratios (MAG:PLP1 and PDGFRβ:CD31) but for measurements of individual proteins we calculated *Z*-scores to allow us to combine the datasets from the two brain banks.

We used SPSS version 21 (SPSS, Chicago, IL, USA)) and GraphPad Prism version 8 (GraphPad Software, La Jolla, CA, USA) for statistical analyses. Marker level differences between groups subdivided according to BS were analysed using either one-way or two-way ANOVA, as appropriate, with Dunnet’s *post hoc* multiple comparison testing; for these analyses, BS0–II samples served as controls. Pearson’s or Spearman’s test was used to assess linear or rank-order correlation, as appropriate. *P*-values < 0.05 were considered statistically significant.

## Results

### Widespread markers of cerebrovascular dysfunction associated with cerebral hypoperfusion in early AD

**Figure 1:**
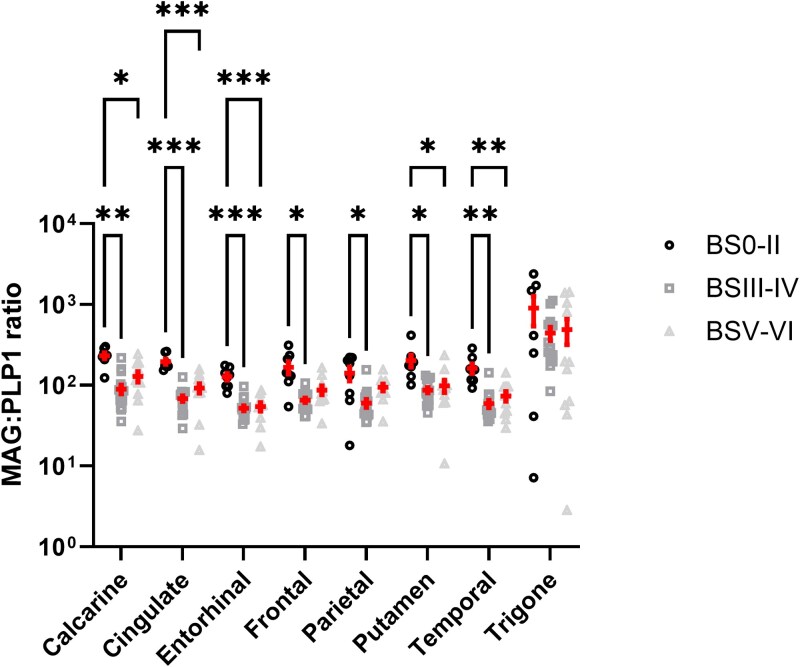
**Biochemical evidence of widespread cerebral hypoperfusion in early stage/intermediate pathology Alzheimer’s disease (AD).** The MAG:PLP1 ratio, which declines in chronic brain ischaemia, is lower across all regions in early-stage/intermediate pathology AD cases. Each data-point represents the average of duplicate measurements in a single brain region. Mean ± standard error of the mean are shown. *P < 0.05, **P < 0.01, ***P < 0.001. BS = Braak tangle stage.

We previously showed that the ratio of MAG:PLP1 was lower in BSIII–IV than BS0–II controls in homogenates of the parietal cortex, consistent with chronic hypoxic ischaemia in early-stage AD.^24^ In this study, MAG:PLP1 was lower in BSIII–IV than BS0–II controls in all cortical regions studied, as well as the putamen: cingulate and entorhinal cortex (both *P* < 0.001); temporal and calcarine cortex (both *P* < 0.01); and frontal and parietal cortex and putamen (all *P* < 0.05) (Fig. 1). MAG:PLP1 remained lower in BSV–VI than BS0–II in the cingulate and entorhinal cortex (*P* < 0.001), temporal cortex (*P* > 0.01), calcarine cortex and putamen (*P* < 0.05) but did not differ significantly between BSIII–IV and BSV–VI brains in most regions. The relative reduction in the MAG:PLP1 ratio in the trigonal white matter in higher BS brains relative to BS0–II did not reach statistical significance. A summary of the MAG:PLP1 ratio within groups stratified by Braak tangle stage is shown in Supplementary Table 3.

A similar trend towards lower MAG:PLP1 was observed across all regions when cases were stratified according to Thal phase but significance was reached only for the calcarine and cingulate cortex, and putamen. The MAG:PLP1 ratio was unchanged when cases were stratified for CAA severity (CAA 0 versus 1 versus 2–3) or cSVD severity (cSVD 0 versus 1 versus 2–3), across all brain regions. These data are shown in Supplementary Table 3.

**Figure 2:**
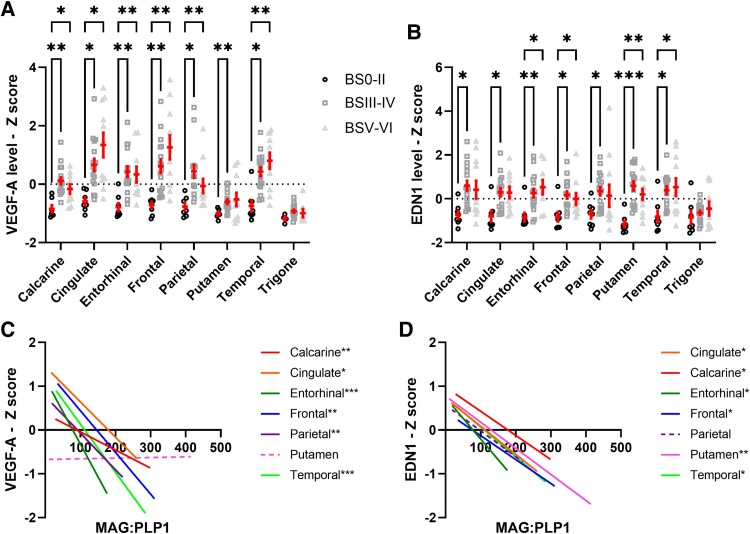
**Elevated VEGF-A and EDN1 associated with cerebral hypoperfusion in early-stage Alzheimer's disease.** Concentrations of (**A**) VEGF-A and (**B**) EDN1, measured by sandwich ELISA, in Braak tangle stage groups BS0–II, BSIII–IV and BSV–VI, across brain regions. Each data-point represents the average of a duplicate measurement in a single brain region. *Z*-scores were calculated to combine data from two independent cohorts. Mean ± standard error of the mean are shown. Correlation analysis between (**C**) VEGF-A and (**D**) EDN1 with the MAG:PLP1 ratio in the different brain regions. Solid linear regression lines are used to indicate brain regions with significant correlations, dashed lines indicate brain regions with non-significant relationships. Trigone is not shown as it did not fit within the range of the graph. *P < 0.05, **P < 0.01, ***P < 0.001 indicate statistical strength of correlations within respective brain regions.

The concentration of VEGF-A, a more acute marker of cerebral ischaemia, was significantly higher in homogenates of grey matter regions from BSIII–IV than BS0–II brains: the entorhinal, frontal and calcarine cortex and putamen (all *P* < 0.01) and the temporal, cingulate and parietal cortex (all *P* < 0.05) (Fig. 2A). Concentrations of EDN1 were also higher in homogenates from grey matter regions in BSIII–IV and BSV–VI brains relative to BS0–II (Fig. 2B). VEGF-A and EDN1 concentrations in the trigone were lower and did not differ in relation to BS. Regional comparisons of VEGF-A and EDN1 levels after stratifying the cohort by Braak tangle stage, Thal phase, CAA or SVD severity are shown in Supplementary Table 4. VEGF-A and EDN1 levels varied significantly according to Braak tangle stage in most brain regions except for the putamen and trigone (EDN1), and parietal cortex and trigone (VEGF-A). VEGF-A varied significantly according to Thal phase in the calcarine cortex only. There was a trend towards higher VEGF-A with increasing CAA and cSVD severity; this reached statistical significance for SVD severity within the putamen only.

VEGF-A concentrations correlated inversely with the MAG:PLP1 ratio in all grey matter regions apart from the putamen (Fig. 2C). EDN1 correlated inversely with MAG:PLP1 in most grey matter regions except for the parietal cortex, and not in trigonal white matter (Fig. 2D). The correlation co-efficient and *P*-values for the relationships between MAG:PLP and markers of cerebrovascular function, including VEGF-A and EDN1, are shown in Supplementary Table 8A. Additional correlation analysis within individual Braak stage groups is shown in Supplementary Table 8B.

**Figure 3:**
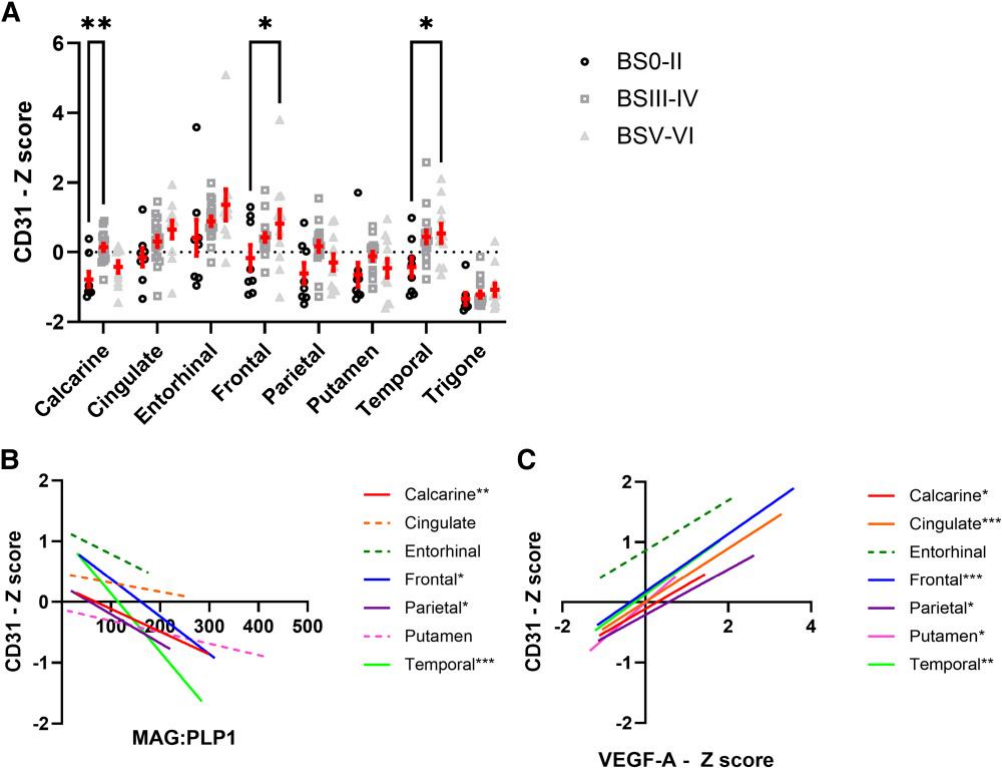
**CD31 levels are elevated in early-stage Alzheimer's disease and are related to MAG:PLP1 and VEGF-A**. (**A**) CD31 levels, measured by ELISA, in Braak tangle stage groups BS0–II, III–IV and BSV–VI in the different brain regions. Each data-point represents the average of a duplicate measurement in a single brain region. *Z*-scores were used to combine data from two independent cohorts. (**B** and **C**) Correlation analysis between CD31 and VEGF-A, and MAG:PLP1 ratio, in the different brain regions. Solid linear regression lines are used to indicate brain regions with significant correlations, dashed lines indicate brain regions with non-significant relationships. Trigone is not shown as it did not fit within the range of the graph. *P < 0.05 and **P < 0.01 indicate statistical strength of correlations within respective brain regions.

CD31, a marker of endothelial cells and microvessel density, was generally elevated across most brain regions in early-stage/intermediate pathology AD; however, significant differences were found only in calcarine, for BSIII–IV relative to BS0–II (*P* < 0.01), and in the frontal cortex and putamen, for BSV–VI relative to BS0–II (both *P* < 0.05) (Fig. 3A). Regional levels of CD31 related to Braak stage, Thal phase, and CAA and SVD severity are shown in Supplementary Table 5. CD31 levels did not vary according to Thal phase except within the trigone, or according to CAA or cSVD severity.

CD31 correlated inversely with MAG:PLP1 in the temporal cortex (*r* = 0.62, *P* < 0.001) and trigonal white matter (*r* = 0.53, *P* < 0.01) and weakly in the calcarine, frontal and parietal cortex (Fig. 3B). The correlation co-efficient and *P*-values are shown in Supplementary Table 8. CD31 concentrations correlated positively with VEGF-A across all brain regions: the correlations were strongly positive within the cingulate, frontal and temporal cortex, and the trigone. Weaker but significant correlations were observed in the putamen, parietal and calcarine cortex, and approaching significance in the entorhinal cortex (Fig. 3C). The correlation coefficient and *P*-values are shown in Supplementary Table 9. Weak but significant correlations were also observed between CD31 and EDN1 in the calcarine cortex (*r* = 0.51, *P* < 0.01) and cingulate cortex (*r* = 0.43, *P* < 0.05) and between EDN1 and VEGF-A in calcarine cortex (*r* = 0.40, *P* < 0.05) and entorhinal cortex (*r* = 0.41, *P* < 0.05) (Supplementary Table 9).

### EDN1 correlates with Aβ_1–42_ in low-pathology controls

The levels of Aβ_1–42_, Aβ_1–40_ and ptau-231, in relation to BS and brain region, are summarized in Supplementary Fig. 1. Aβ_1–42_ and Aβ_1–40_ levels were higher in BSIII–IV and BSV–VI than in BS0–II brains in most cortical regions. Concentrations of ptau-231 were significantly higher in BSV–VI than BS0–II in most regions. Aβ_1–42_, Aβ_1–40_ and ptau-231 levels correlated with each other in most brain regions (Supplementary Table 10).

**Figure 4:**
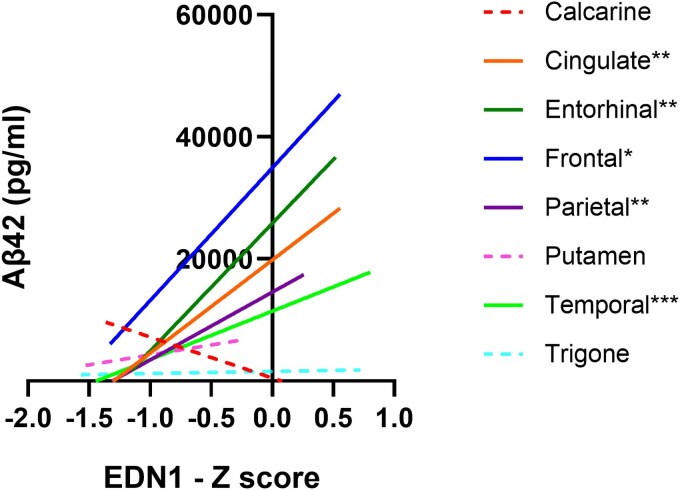
**EDN1 correlates positively with Aβ_1–42_ in early-stage Alzheimer's disease.** Linear regression and 95% confidence intervals are shown for EDN1 and Aβ_1–42_ in the BS0–II group in all brain regions. Solid linear regression lines are used to indicate brain regions with significant correlations, dashed lines indicate brain regions with non-significant relationships. *P < 0.05, **P < 0.01, ***P < 0.001 indicate statistical strength of correlations within respective brain regions. Aβ = amyloid-β; BS = Braak tangle stage.

We performed correlation analysis between MAG:PLP1, VEGF-A, EDN1 and CD31 with Aβ_1–42_, Aβ_1–40_ and ptau-231. MAG:PLP1 did not correlate strongly with Aβ_1–42_, Aβ_1–40_ or ptau-231 in any brain region. VEGF-A, EDN1 and CD31 did not correlate with pathological markers across the entire cohort but analysis within groups stratified according to BS stage revealed that EDN1 correlated strongly with Aβ_1–42_ in the BS0–II group in almost all cortical regions: cingulate (*r* = 0.96, *P* < 0.01), entorhinal (0.932, *P* < 0.01), temporal (*r* = 0.839, *P* < 0.001), parietal (*r* = 0.878, *P* < 0.01) and frontal cortex (*r* = 0.717, *P* < 0.05) (Fig. 4; correlation coefficient and *P*-values are shown in Supplementary Table 11).

### Angiogenic protein expression is dysregulated in early-stage AD

We used an angiogenesis proteome profiler assay to compare the concentrations of 55 proteins associated with angiogenesis across the three Braak tangle stage subgroups in homogenates from a subset of temporal cortex samples. We focused on the temporal cortex as many of the relationships reported above were consistently observed within this brain region.

**Figure 5:**
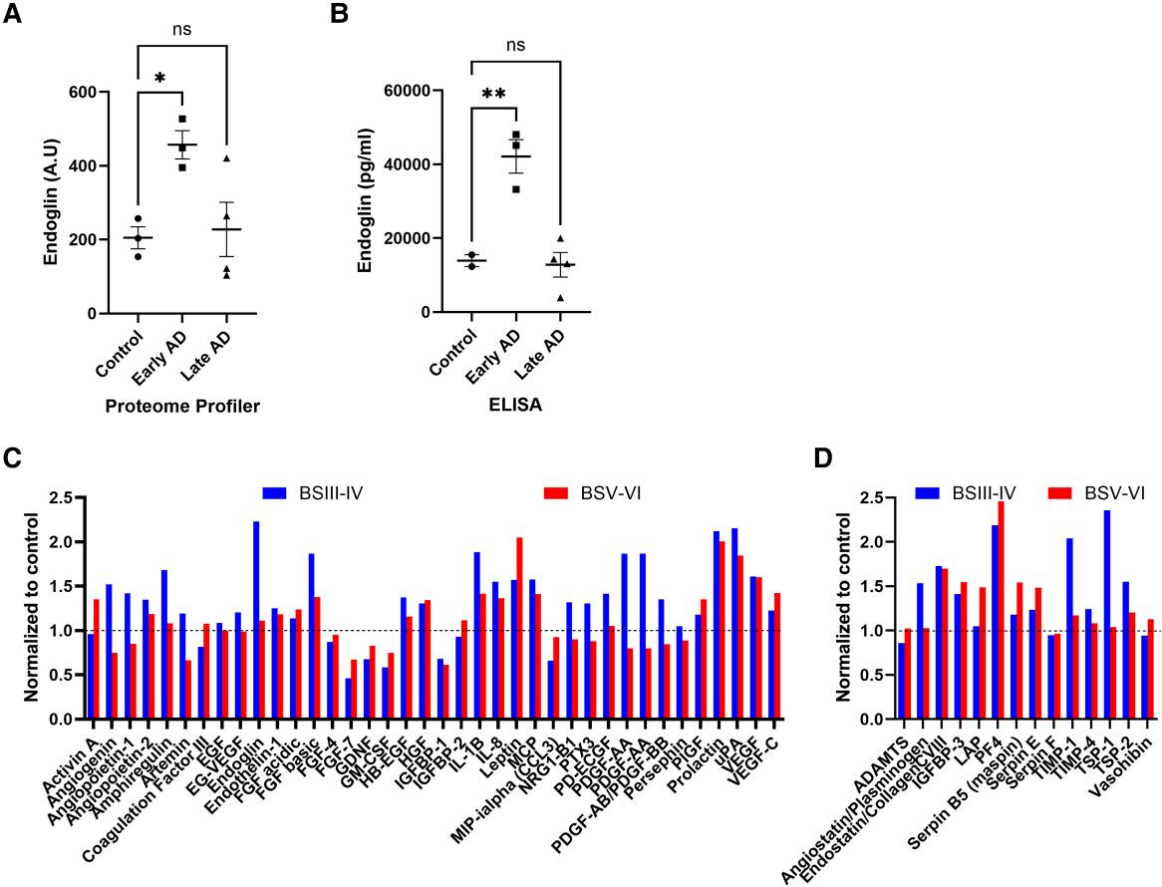
**Mediators of angiogenesis are dysregulated in early-stage/intermediate pathology Alzheimer's disease.** (**A** and **B**) Levels of endoglin (CD105), a marker of neoangiogenesis, measured first using the angiogenesis proteome profiler and then independently by commercial ELISA kit. Mean and standard error of the mean are shown. *P < 0.05, **P < 0.01. (**C** and **D**) Levels of pro-angiogenic (**C**) and anti-angiogenic (**D**) mediators in early-stage/intermediate pathology [Braak tangle stage (BS) III–IV] (blue) and late-stage AD (BSV–VI) (red), relative to the levels in minimal pathology BS0–II cases.

There was a striking increase in endoglin (CD105), a marker of neoangiogenesis upregulated in proliferating endothelial cells (reviewed in ten Dijke *et al*.^31^), in BSIII–IV (*P* < 0.05, one-way ANOVA) (Fig. 5A). We confirmed the validity of this finding by independently measuring levels of endoglin in the same samples by sandwich ELISA and showed a significantly higher endoglin level in BSIII–IV than BS0–II brains (*P* < 0.01, one-way ANOVA) (Fig. 5B).

We next compared the levels of 55 angiogenic proteins in relation to Braak tangle stage. The relative expression levels of angiogenic proteins in BSIII–IV and BSV–VI normalized to BS0–II controls (set to 100%) are shown in Fig. 5C and D. Proteins generally regarded as pro-angiogenic are grouped together and shown in Fig. 5C and those considered to have anti-angiogenic properties are grouped and shown in Fig. 5D. Differences between Braak tangle stage groups in the mean relative level of each marker were assessed by two-way ANOVA with Dunnet’s multiple comparisons test (Supplementary Table 12). The relative levels of several angiogenic proteins, including EDN1, VEGF-A, endoglin and others such as angiogenin, angiopoietein-2, angiostatin, peaked in BSIII–IV, but due to the relatively small cohort size, most did not reach statistical significance. Those proteins that reached significance included fibroblast growth factor (FGF)-basic, which was significantly higher in BSIII–IV than BS0–II (*P* < 0.0001), and FGF-acidic protein which was higher in BSV–VI than BS0–II (*P* < 0.0001). Matrix metalloproteinase (MMP)-8, MMP-9 and platelet factor 44 were significantly higher in BSIII–IV than BS0–II, and more so in BSV–VI. A few markers, such as coagulation factor III (*P* < 0.01), FGF-7 and insulin-like growth factor-binding protein 1 (IGF-BP1)-1 (*P* = 0.1), were lower in either BSIII–IV or BSV–VI than BS0–II controls.

### PDGFRβ levels are lower in early-stage AD and correlate with MAG:PLP1 and amyloid-β_1–42_

**Figure 6:**
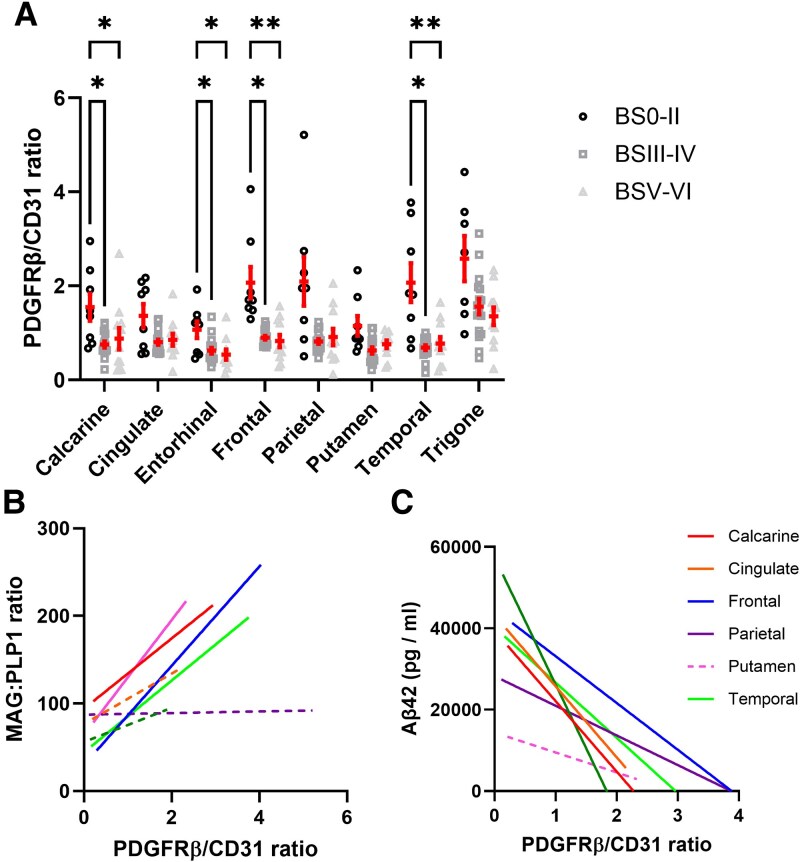
**Widespread reduction in PDGFRβ related to cerebral hypoperfusion and Aβ_1–42_ in early-stage Alzheimer's disease.** (**A**) Levels of pericyte marker PDGFRβ relative to endothelial marker CD31 (i.e. PDGFRβ:CD31), in the different brain regions. Each data-point represents the ratio for an individual. Mean ± standard error of the mean are shown. (**B** and **C**) Correlation analysis between the PDGFRβ:CD31 and MAG:PLP1 ratio, and PDGFRβ:CD31 ratio and Aβ_1–42_, in the different brain regions. Solid linear regression lines are used to indicate brain regions with significant correlations, dashed lines indicate brain regions with non-significant relationships. Trigone is not shown as it did not fit within the range of the graph. *P < 0.05, **P < 0.01, ***P < 0.001. Aβ = amyloid-β; BS = Braak tangle stage.

We next measured PDGFRβ levels and calculated the PDGFRβ:CD31 ratio as an index of pericyte content relative to changes in endothelial cell and microvessel density. The regional PDGFRβ:CD31 ratio in relation to Braak tangle stage, Thal phase, CAA and SVD is shown in Supplementary Table 6. PDGFRβ:CD31 was lower in BSIII–IV than BS0–II in all grey matter regions (*P* < 0.05) (Fig. 6A). PDGFRβ:CD31 correlated positively with MAG:PLP1 in the frontal (*r* = −0.69, *P* < 0.01) and temporal cortex (*r* = −0.60, *P* < 0.001), and more weakly in the calcarine cortex and putamen (Fig. 6B). PDGFRβ:CD31 correlated inversely with Aβ_1–42_ concentration in all grey matter regions (Fig. 6C) and correlated inversely with VEGF-A and EDN1 in most brain regions. The correlation coefficient and *P*-values are shown in Supplementary Table 13.

### Fibrinogen, a marker of BBB leakiness, is higher in late-stage AD and correlates strongly with VEGF-A, CD31 and ptau-231

**Figure 7:**
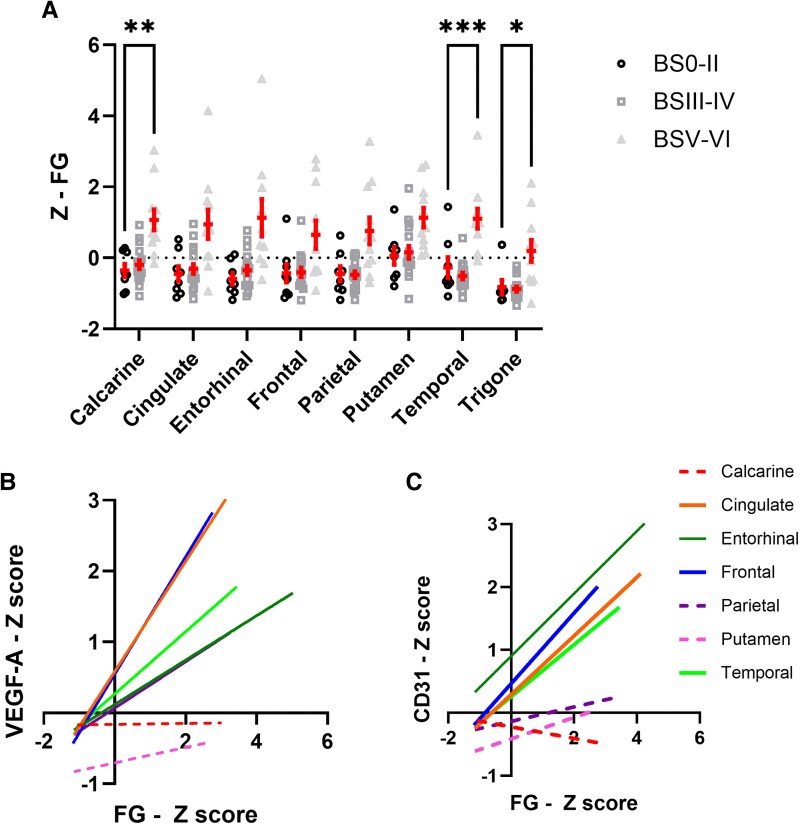
**Widespread blood–brain barrier (BBB) leakage in late-stage Alzheimer's disease.** (**A**) Bar charts showing fibrinogen level, a proxy marker of BBB leakiness in relation to Braak tangle stage BS0–II, III–IV and V–VI, in the different brain regions. *Z*-scores were calculated to combine data from two independent cohorts. Each data-point represents an individual. Mean ± standard error of the mean are shown. (**B** and **C**) Correlation between fibrinogen (FG) level, VEGF-A and CD31 across regions. Solid linear regression lines are used to indicate brain regions with significant correlations, dashed lines indicate brain regions with non-significant relationships. Trigone is not shown as it did not fit within the range of the graph. *P < 0.05, **P < 0.01, ***P < 0.001.

Fibrinogen levels in relation to Braak tangle stage, Thal phase, CAA and SVD across brain regions is shown in Supplementary Table 7. Higher fibrinogen levels in brain tissue are a proxy marker of BBB leakiness. Concentrations of fibrinogen were higher in BSV–VI than BS0–II in the temporal, calcarine, cingulate and entorhinal cortex and in the trigone (Fig. 7A). A similar trend was found in the frontal and parietal cortex, and putamen, did not reach statistical significance. In most cortical regions, fibrinogen concentrations correlated strongly with VEGF-A and CD31 (Fig. 7B and C). Fibrinogen concentrations did not correlate with Aβ_1–42_ or Aβ_1–40_, but correlated positively with the concentration of ptau-231 in most brain regions: the temporal and parietal cortex, trigone and approaching statistical significance in the calcarine and cingulate cortex. The correlation coefficient and *P*-values are shown in Supplementary Table 14.

We explored the regional relationships between fibrinogen concentrations and vascular markers after stratifying the cohort according to Braak tangle stage. Positive correlations between fibrinogen and VEGF-A, and between fibrinogen and CD31, were observed in late-stage (BSV–VI) brains only (Supplementary Table 15).

Several of the angiogenic proteins assessed with the angiogenic proteome profiler assay correlated positively with the concentration of fibrinogen: latency-associated peptide (LAP, the pro-peptide of TGF-beta-1), leptin and prolactin, with serpins B and E approaching significance. IGF-BP1 correlated inversely with fibrinogen levels (Supplementary Fig. 2).

## Discussion

Cerebral hypoperfusion and BBB leakiness contribute to cognitive decline and are related to disease pathogenesis in AD.^32-34^ The pathophysiological pathways and processes that mediate cerebrovascular dysfunction and their relationship to the build-up and regional spread of Aβ and tau in AD remain unclear. In the present study, we used well-validated post-mortem biochemical markers to investigate the regional relationships between cerebrovascular dysfunction and Aβ and tau accumulation in AD. We studied multiple cortical brain regions and the putamen and trigonal white matter, from brains with widely differing levels of AD pathology. The brains were selected to have minimal confounding vascular or non-AD neurodegenerative pathologies. We present evidence of widespread cerebral hypoperfusion (lower MAG:PLP1 ratio), neoangiogenesis (higher endoglin) and vascular remodelling (higher CD31 and lower PDGFRβ levels) associated with higher VEGF-A and EDN1 expression in early-stage AD. Elevated EDN1 and reduced PDGFRβ were both strongly related to Aβ_42_ concentration in early AD. In contrast, fibrinogen concentration, indicative of BBB leakiness, was highest in late-stage AD and was associated with VEGF-A, MAG:PLP1 and ptau-231. Cerebrovascular markers were dysregulated in relation to Braak tangle stage, rather than Thal phase, and were not strongly influenced by CAA or cSVD severity.

We previously demonstrated lower MAG:PLP1 within the precuneus in early-stage/intermediate pathology sporadic AD cases.^24,25^ The present study reveals that pathological changes associated with evidence of impaired tissue oxygenation, i.e. resulting from cerebral hypoperfusion, are much more widespread, affecting all of the grey matter regions that we analysed. This is consistent with previous findings from clinical studies showing widespread reduction in regional blood flow in AD. A meta-analysis of 244 clinical studies of 13 664 participants revealed a reduction in resting blood flow within the posterior cingulate, frontal, temporal and parietal lobes, including the precuneus, in mild cognitive impairment (MCI), and a more widespread regional reduction in AD.^35^ Another meta-analysis specifically in MCI, that incorporated 13 arterial spin labelling-MRI studies comparing 495 MCI subjects with 441 healthy controls, also reported widespread cerebral blood flow reduction in the precuneus, inferior parietal lobule, superior occipital gyrus, middle temporal gyrus and middle occipital gyrus in MCI.^36^ In familial AD, where the age of onset can be predicted with a high degree of certainty, a reduction in blood flow was first demonstrable within the precuneus 10–15 years before the predicted onset of cognitive decline, with subsequent spread of cerebral hypoperfusion overlapping with the distribution of Aβ plaque accumulation in the cortex.^7^ In a cohort of 187 individuals with subjective cognitive decline, low cerebral blood flow at baseline was associated with steeper cognitive decline and elevated PET-Aβ load.^37^ As in our previous study of the precuneus, in most regions the MAG:PLP1 ratio remained stable or increased slightly in BSV–VI compared with BSIII–IV, probably reflecting a drop in metabolic demand as a consequence of synaptic loss and neurodegeneration in late-stage AD.

We previously reported on the pathophysiology of cerebral hypoperfusion in AD. Across independent cohorts, we showed that a reduction in MAG:PLP1 correlated inversely with VEGF-A in multiple cortical brain regions, including the frontal, cingulate and parahippocampal cortex^26^ and precuneus.^24,25^ The fall in MAG:PLP1 and the rise in VEGF-A correlated with increased EDN1, a potent vasoconstrictor peptide^24,26^ that is upregulated in neuroblastoma cells by Aβ_1–42_,^27^ and in human brain endothelial cells by both Aβ_1–40_ and Aβ_1–42_ (but particularly the former).^38^ In the present study, VEGF-A and EDN1 levels were highest in early-stage AD, in all cortical regions as well as the putamen, and correlated inversely with MAG:PLP1. Stratification of the cohort according to Braak tangle stage revealed strong positive correlations between EDN1 and Aβ_1–42_ (but not Aβ_1–40_ or ptau-231) in homogenates from BS0–II brains, in keeping with other evidence that Aβ_1–42_-induced production of neuronal or microglial EDN1 is an upstream driver of cerebral hypoperfusion. Physiological levels of oligomeric Aβ_1–42_ were recently shown to induce EDN1-mediated pericyte constriction of capillaries, via oxidative stress signals, in rodent models and in human brain cortex slices.^10^ Together these data suggest that EDN1-mediated vasoconstriction contributes to cerebral hypoperfusion in the very early stages of AD, making this a promising target for therapeutic intervention (reviewed in Palmer and Love^39^).

Concentrations of CD31, a marker of endothelial cells, were raised in BSIII–IV in most brain regions and correlated inversely with MAG:PLP1 and positively with VEGF-A. To explore whether this reflected neoangiogenesis in early-stage AD, we measured endoglin levels, a protein which is upregulated in proliferating endothelial cells.^31^ Endoglin was significantly higher in BSIII–IV than BS0–II, but similar to controls in BSV–VI. This may indicate a biphasic angiogenic response to cerebral hypoperfusion in early-stage AD, which becomes defective in late-stage AD. To explore this further, we compared the relative expression of a range of angiogenesis-related proteins within the temporal cortex and found that several angiogenic mediators or regulators were also elevated in early-stage AD. These included angiopoietin-2 and acidic and basic FGF. Differential upregulation of pro-angiogenic endothelial gene transcripts (*ANGPT2*, *HIF1α*, *MEF2C* and *FGF2*) and downregulation of downstream angiogenic effectors (*VEGFR2*, *EGFR*, *TGFβ*), identified by single-nuclei molecular mapping of the cerebral vasculature in AD,^20^ provide mechanistic clues pointing towards an impaired angiogenic response in AD. Other markers, such as thrombospondin-1, are induced in endothelial senescence, which may also contribute to vascular remodelling, BBB leakiness and tau pathology in AD.^40,41^

Pericytes play an essential role in regulating angiogenesis and BBB permeability; pericyte degeneration and BBB leakiness have been associated with cognitive decline, in ‘preclinical’ AD.^18,19^ We previously demonstrated lower levels of the pericyte protein PDGFRβ within the precuneus in AD, correlating inversely with the level of brain tissue fibrinogen, a marker of BBB leakiness.^24,25^ Membrane shedding of PDGFRβ from injured pericytes may account for reduced PDGFRβ in brain tissue and subsequent elevated levels of soluble PDGFRβ fragments in CSF, as reported in AD.^33^ In the present study, PDGFRβ concentrations, normalized to CD31 to account for variations in microvessel content, were reduced in early-stage AD in most cortical brain regions. In grey matter regions, PDGFRβ:CD31 correlated with MAG:PLP1, extending our previous findings.^25,30^ The decline in PDGFRβ:CD31 was also associated with higher Aβ_1–42_ but not Aβ_1–40_ or ptau-231 in early-stage AD. Soluble oligomeric and fibrillar Aβ_1–42_ are toxic to pericytes *in vitro*,^42,43^ whereas soluble Aβ_1–40_ may be protective.^44^ These data highlight cerebral hypoperfusion and Aβ_1–42_ as likely contributors to pericyte degeneration in the early stages of AD.

In the present study, the fibrinogen level in brain tissue homogenates was elevated within most brain regions but mainly within late-stage BSV–VI AD cases. Fibrinogen levels correlated strongly with VEGF-A and CD31 implicating neovascularization as an important mediator of BBB breakdown in AD, as suggested in previous studies.^45-47^ There was weak evidence that fibrinogen levels were related to either Aβ_1–42_ or Aβ_1–40_ but fibrinogen concentrations correlated strongly with ptau-231 in most cortical brain regions. These findings build upon recent studies highlighting a close relationship between tau and cerebrovascular dysfunction. For example, vascular mural cells interact with and phagocytose tau,^48^ and tau accumulates around cortical arterioles,^11^ perhaps reflecting impaired local vascular clearance or degradation. In mouse models, overexpression of tau induces a range of cerebrovascular abnormalities, including morphologically abnormal forms of neoangiogenesis^12,49,50^ and endothelial senescence.^40^ Investigating the role of tau in endothelial senescence in relation to BBB leakiness warrants further investigation. Our angiogenic profiler data identified a relationship between fibrinogen, MMP-8 and MMP-9, which was strongest in late-stage AD. Other less recognized mediators of BBB leakiness, such as leptin, prolactin and IGF-BP1, also correlated with fibrinogen, highlighting potential novel pathways associated with BBB regulation that warrant further investigation.

Our study has limitations. The number of low-pathology controls was relatively small and most controls had some form of minor pathology; inclusion of more controls including some with entirely absent pathology would be informative in future studies. Measuring the levels of vascular and pathological markers in brain tissue samples by ELISA allows for a much greater volume of tissue to be sampled, and a large panel of markers can be measured in the same tissue; however, this approach does not offer insights into alterations in the cell-type-specific expression of vascular mediators or markers. Spatial transcriptomics, multiplex spatial immunohistochemistry or biochemical evaluation of vessel-enriched preparations would provide further insights into the contribution of different vascular and non-vascular cells to cerebrovascular dysfunction in AD. In a human post-mortem study such as this, associations and correlations may provide circumstantial evidence in support of hypotheses but cannot prove causality. Lastly, the presence of co-morbidities such as such as hypertension, diabetes and factors relating to metabolic syndrome, which may contribute to vascular dysfunction independently of ADNC, are inconsistently reported or unavailable preventing the assessment of these potentially important confounders in this study.

In conclusion, our study provides evidence of widespread cerebral hypoperfusion and dysregulated angiogenesis associated with vascular remodelling and BBB leakiness that begins in the early stages of AD and persists in later disease. We highlight Aβ_1-42_-EDN1-induced cerebral hypoperfusion, VEGF-A-induced vascular remodelling and dysregulated angiogenic signalling, as important contributors to cerebrovascular dysfunction and BBB leakiness beginning in the early stages of AD. A reduction in the pericyte protein, PDGFRβ, related to lower MAG:PLP1 and elevated Aβ_1–42_ in BSIII–IV, and elevated fibrinogen levels indicative of BBB leakiness in late-stage AD, may also reflect vascular remodelling in AD. Lastly, we highlight several points along this EDN1-hypoperfusion-VEGF-neoangiogenesis continuum that may be amenable to therapeutic intervention in the early stages of AD.

## Supplementary Material

awaf394_Supplementary_Data

## Data Availability

The data underlying this article will be shared on reasonable request to the corresponding author. All cases used in this study can be identified by a unique UK Brain Bank identifier number shown in Supplementary Table 16 allowing researchers to request brain tissue samples from the same donors.
