# Supplementary material for: Post-mortem evidence of pathogenic angiogenesis and abnormal vascular function in early Alzheimer’s disease
Source: Brain. 2025 Oct 22;149(4):1182–93. doi: 10.1093/brain/awaf394 (PMC13058453; doi:10.1093/brain/awaf394)
Supplement: awaf394_Supplementary_Data [file awaf394_supplementary_data.pdf]

Supplementary Table 1. Study demographic and neuropathology

Low Pathology controls:

ID	Age	Sex	PM	CERAD	CERAD np	B S	Thal	CA AF	CA AT	CA AO	CAA Total	SVD F	SVD T	SVD P	SVD O
1	93	M	34.5	No AD	None	2	1	0	0	0	0	0-1	0	1	1
2	75	M	46.5	No AD	None	2	4	0	0	0	0	0	0	0	0
3	92	F	71	No AD	None	2	4	3	1	0	1	0	0	0	0
4	87	F	29.5	Possible	Sparse	2	4	1	0	2	1	0	0	0	0
5	81	F	42	No AD	None	0	0	0	0	-	-	0	-	-	0
6	80	M	58	No AD	None	1	0	0	0	-	-	1	-	-	1
7	69	F	48	No AD	Sparse	0	1	1	1	-	-	0	-	-	0
8	95	M	18	No AD	Sparse	2	5	2	3	-	-	2	-	-	1
Av	*84.0*	*4M*	*43.4*			*1.4*	*2.4*	*0.9*				*0.4*			
SD	*9.3*	*4F*	*16.6*			*0.9*	*2.1*	*1.1*				*0.7*			

Intermediate Pathology AD:

ID	Age	Sex	PM	CERAD	CERAD np	B S	Thal	CA AF	CA AT	CA AO	CAA Total	SVD F	SVD T	SVD P	SVD O
9	77	M	41	Possible	Sparse	3	4	0	0-2	2	2	1-3	1	1	1
10	97	F	86	Probable	Moderate	4	4	0	0	0	0	2-3	2	2	-
11	91	F	74	Probable	Frequent	4	4	0	0	2	1	2-3	2	1	1
12	95	F	88	Possible	Moderate	4	4	1	1	1	1	2	1	2	1
13	79	M	76	Probable	Moderate	3	4	3	0-2	1	2	2	1	1	1
14	94	F	16	Possible	Sparse	3	3	0	0	0	0	0-1	0-1	1	0
15	86	M	35	Possible	Frequent	4	4	0	0	0	0	0-1	0	1	0
16	97	F	15	Possible	Sparse	3	4	3	2	1	2	0-1	0-1	0	0
17	90	M	31	Possible	Sparse	3	1	1	0	2	1	0-1	1-2	2	0
18	73	M	22	Probable	Sparse	3	4	2	2	3	2	0	0	0	0
19	86	M	56	Probable	Moderate	4	3	1	2-3	3	3	0-1	0	0	0
20	92	M	47	Probable	Moderate	4	5	1	1	2	1	1	0	1	1
21	75	M	105	Possible	Sparse	3	5	0	0	0	0	0-1	0	0	0
22	98	M	49	Possible	Sparse	3	5	0	0	0	0	0	0	0	0
23	81	M	38	Definite	Frequent	4	5	0	0	0	0	0 -1	0	0	0
24	93	M	37	Probable	Moderate	4	3	0	0	0	0	0	0-1	0	1
25	83	F	41.75	Definite	Frequent	3	5	2	2	2	2	0	0	1	0
Av	*87.8*	*11M*	*51.0*			*3.5*	*4.0*	*0.8*				*0.7*			
SD	*8.4*	*6F*	*27.3*			*0.5*	*1.1*	*1.1*				*0.4*			

Late-stage Pathology AD:

ID	Age	Sex	PM	CERAD	CERAD np	B S	Thal	CA AF	CA AT	CA AO	CAA Total	SVD F	SVD T	SVD P	SVD O
26	72	F	11.5	Definite	Frequent	6	5	3	3	2	3	0	0	0	1
27	86	F	37.5	Definite	Frequent	6	5	0	1	1	1	0-1	1	1	1
28	64	M	44.75	Definite	Frequent	6	5	3	2	3	2	0	0	0	0
29	90	M	61	Definite	Frequent	5	4	2	2	2	2	0-1	1	1	0
30	72	M	61	Definite	Frequent	6	5	1	1	-	-	0	-	-	2
31	75	M	59	Definite	Frequent	5	5	3	2	-	-	0	-	-	0
32	93	M	36	Intermed	Moderate	5	2	1	2	-	-	-	-	-	-
33	85	F	35	Definite	Frequent	5	2	3	2	-	-	3	-	-	1
34	79	M	20	Definite	Frequent	6	2	-	-	-	-	-	-	-	-
35	83	F	58	Definite	Frequent	6	2	-	-	-	-	-	-	-	-
36	75	M	36	Definite	Frequent	5	4	3	3	-	-	2	-	-	1
Av	*79.5*	*7M*	*41.8*			*5.6*	*3.7*	*2.1*	*2.0*			*0.8*			
SD	*8.8*	*4F*	*16.8*			*0.5*	*1.4*	*1.4*	*1.0*			*1.0*			

Demographic and neuropathological characteristics of the study cohort. Cases were grouped according to Braak tangle stage into low pathology controls (BS0-II) (n = 8), intermediate- (BSIII-IV) (n = 17) and late-stage (BSV-VI) pathology AD. Sex – M = Male, F = Female. PM = post-mortem delay (hours). Clinical diagnosis based on Consortium to Establish a Registry for Alzheimer's Disease (CERAD): No Alzheimer's (No AD), possible, probable and definite. CERAD Neuropathology (np) score to assess the density of neuritic plaques: none, sparse, moderate and frequent. BS = Braak tangle stage groups: 0-II, III-IV and V-VI (but shown here in Arabic numerals, for convenience). Thal = Thal phase (0-6). All cases included in the study were Braak Lewy body stage 0 (not shown). CAA = cerebral amyloid angiopathy score based on the method of Olichney[1]: 0 for vessels devoid of Aβ, 1 for limited deposits of vascular amyloid, 2 for the circumferential deposition of Aβ in several vessels, and 3 for severe and extensive CAA, assessed in frontal (F), temporal (T), and occipital (O) lobe and total score. SVD = cerebral small vessel disease score, specifically related to arteriolosclerosis and excluding CAA, based on a four-point semi-quantitative scale according to the level of arteriolar wall thickening and associated narrowing of vessel lumens[2]: 0 for normal vessel wall thickness, 1 for slightly increased thickness, 2 for moderately increased thickness, and 3 for markedly increased thickness with concomitant reduction in many arteriolar lumina to less than 50% of the outer diameter of the vessel, assessed in the frontal, temporal, parietal and occipital lobe. – denotes missing information. A summary of the mean (Av) and standard deviation (SD) for age-at-death, PM delay, Braak tangle and Thal stage, and CAA and SVD score in the frontal lobe is shown in italics for each of the groups.

References

1. Olichney JM, Hansen LA, Hofstetter CR, Lee JH, Katzman R, Thal LJ. Association between severe cerebral amyloid angiopathy and cerebrovascular lesions in Alzheimer disease is not a spurious one attributable to apolipoprotein E4. Arch Neurol. 2000;57(6):869-74.

2. Barker R, Wellington D, Esiri MM, Love S. Assessing white matter ischemic damage in dementia patients by measurement of myelin proteins. J Cereb Blood Flow Metab. 2013;33(7):1050-7.

Supplementary Table 2. Demographic and neuropathological features of the sub-cohort used to assess angiogenic signalling in the temporal cortex

ID:	Age	Sex	PM	CERAD	CERAD np	BS:	Thal Phase:	CAA-F:	CAA-T	CAA-O	CAA-Total	SVD-F	SVD-T	SVD-P
1	93	M	34.5	No AD	None	2	1	0	0	0	0	0-1	0	1
2	75	M	46.5	No AD	None	2	4	0	0	0	0	0	0	0
3	92	F	71	No AD	None	2	4	3	1	0	1	0	0	0
Av	*86.67*		*50.67*			*2.00*	*3.00*	*1.00*	*0.33*	*0.00*	*0.33*	*0.00*	*0.00*	*0.33*
SD	*10.12*		*18.60*			*0.00*	*1.73*	*1.73*	*0.58*	*0.00*	*0.58*	*0.00*	*0.00*	*0.58*
9	77	M	40.5	Possible	Sparse	3	5	0	0 to 2	2	2	1 to 3	1	1
4	87	F	29.5	Possible	Sparse	2	4	1	0	2	1	0	0	0
25	83	F	41.75	Definite	Frequent	3	5	2	2	2	2	0	0	1
Av	*82.33*		*37.25*			*2.67*	*4.67*	*1.00*	*1.00*	*2.00*	*1.67*	*0.00*	*0.33*	*0.67*
SD	*5.03*		*6.74*			*0.58*	*0.58*	*1.00*	*1.15*	*0.00*	*0.58*	*0.00*	*0.58*	*0.58*
26	72	F	11.5	Definite	Frequent	6	5	3	3	2	3	0	0	0
27	86	F	37.5	Definite	Frequent	6	5	0	1	1	1	0 to 1	1	1
28	64	M	44.75	Definite	Frequent	6	5	3	2	3	2	0	0	0
29	90	M	61	Definite	Frequent	5	4	2	2	2	2	0 to 1	1	1
Av	*80.00*		*47.75*			*5.75*	*4.75*	*2.00*	*2.00*	*2.00*	*2.00*	*0.00*	*0.50*	*0.50*
SD	*14.00*		*12.03*			*0.5*	*0.5*	*1.41*	*0.82*	*0.82*	*0.82*	*0.00*	*0.58*	*0.58*

Demographic and neuropathological characteristics of the sub-cohort used to study angiogenic signalling within the temporal cortex. Cases were grouped into low pathology controls (n = 3), and intermediate (n = 3) and late-stage pathology (n = 4) AD. Sex – M = Male, F = Female. PM = Post-mortem delay (hours). Clinical diagnosis based on Consortium to Establish a Registry for Alzheimer's Disease (CERAD): No Alzheimer's (No AD), possible, probable and definite. CERAD Neuropathology (np) score to assess the density of neuritic plaques: none, sparse, frequent. BS = Braak tangle stage groups: 0-II, III-IV, V-VI. Thal = Thal phase groups: 0-II, III-IV and V-VI. CAA = cerebral amyloid angiopathy score based on method by Olichney[1] : 0 for vessels devoid of Aβ, 1 for limited deposits of vascular amyloid, 2 for the circumferential deposition of Aβ in several vessels, and 3 for severe and extensive CAA, assessed in frontal (F), temporal (T), and occipital (O) lobe. SVD = cerebral small vessel disease score, specifically related to arteriolosclerosis and excluding CAA), based on a four-point semi-quantitative scale according to the level of arteriolar wall thickening and associated narrowing of vessel lumens[2]: 0 for normal vessel wall thickness, 1 for slightly increased thickness, 2 for moderately increased thickness, and 3 for markedly increased thickness with concomitant reduction in many arteriolar lumina to less than 50% of the outer diameter of the vessel, assessed in the frontal, temporal, parietal and occipital lobe. – denotes missing information. A summary of the mean (Av) and standard deviation (SD) for age-at-death, PM delay, Braak tangle and Thal stage, and CAA and SVD score in the frontal lobe is shown in italics for each of the groups. – denotes missing information. The mean (Av) and standard deviation (SD) is shown in italics for age-at-death, PM delay, Braak tangle and Thal stage, and CAA and SVD severity.

References

1. Olichney JM, Hansen LA, Hofstetter CR, Lee JH, Katzman R, Thal LJ. Association between severe cerebral amyloid angiopathy and cerebrovascular lesions in Alzheimer disease is not a spurious one attributable to apolipoprotein E4. Arch Neurol. 2000;57(6):869-74.

2. Barker R, Wellington D, Esiri MM, Love S. Assessing white matter ischemic damage in dementia patients by measurement of myelin proteins. J Cereb Blood Flow Metab. 2013;33(7):1050-7.

Supplementary Table 3: Regional MAG:PLP1 analysis

**Braak stage**		**BS 0-II**			**BS III-IV**			**BS V-VI**		
**Region**	**N**	**Mean**	**SD**	**N**	**Mean**	**SD**	**N**	**Mean**	**SD**	**p-value**
Calcarine	8	232.26	54.03	17	89.39	46.54	11	128.04	62.31	p < 0.0001
Cingulate	8	195.45	41.32	17	68.39	22.24	11	92.06	41.09	p < 0.0001
Entorhinal	8	127.63	34.45	17	51.69	15.76	11	54.35	21.15	p < 0.0001
Frontal	8	166.39	81.31	17	65.15	17.17	11	86.84	36.29	p < 0.0001
Parietal	8	140.54	78.85	17	60.06	28.49	11	94.24	30.89	p = 0.0008
Putamen	8	201.47	94.90	17	86.79	27.80	11	98.21	57.29	p = 0.0001
Entorhinal	8	162.10	63.30	17	58.92	25.27	11	73.23	33.56	p < 0.0001
Trigone	7	900.38	950.79	14	440.37	311.65	11	487.01	561.78	NS
Thal Phase		*Thal 0-2*			*Thal 3-4*			*Thal 5-6*		
Region	*N*	*Mean*	*SD*	*N*	*Mean*	*SD*	*N*	*Mean*	*SD =*	*p-value*
Calcarine	9	191.35	72.89	15	116.18	71.41	12	110.11	67.60	p = 0.025
Cingulate	9	146.85	72.86	15	91.44	51.27	12	87.14	47.87	p = 0.041
Entorhinal	8	95.46	55.34	15	63.07	30.54	12	61.13	29.67	NS (p = 0.09)
Frontal	9	113.88	65.84	15	83.43	36.69	12	93.14	75.09	NS
Parietal	9	146.85	72.86	15	91.44	51.27	12	87.14	47.87	NS
Putamen	9	164.80	101.45	15	108.57	51.89	12	87.99	54.73	p = 0.045
Entorhinal	9	109.60	86.25	15	81.53	42.14	12	74.56	41.41	NS
Trigone	7	326.65	553.15	13	631.26	676.71	12	611.00	489.74	NS
CAA score		*CAA 0*			*CAA 1*			*CAA 2-3*		
Region:	*N*	*Mean*	*SD*	*N*	*Mean*	*SD*	*N*	*Mean*	*SD*	*p-value*
Calcarine	14	128.20	81.41	8	115.69	75.35	12	147.92	80.21	NS
Cingulate	14	109.45	75.27	8	101.57	52.34	12	100.78	54.85	NS
Entorhinal	14	71.11	44.33	8	72.19	46.61	12	68.13	29.97	NS
Frontal	14	88.40	50.70	8	84.43	61.88	12	110.92	70.78	NS
Parietal	14	82.94	59.60	8	81.52	58.02	12	98.00	51.80	NS
Putamen	14	106.48	52.53	8	126.25	118.22	12	119.61	64.25	NS
Entorhinal	14	89.12	57.89	8	93.73	86.20	12	81.13	34.05	NS
Trigone	13	599.95	599.27	6	334.23	197.10	11	705.53	739.59	NS
SVD score		*SVD 0*			*SVD 1*			*SVD 2-3*		
Region	*N*	*Mean*	*SD*	*N*	*Mean*	*SD*	*N*	*Mean*	*SD*	*p-value*
Calcarine	19	128.13	83.86	7	142.09	80.41	7	131.63	76.91	NS
Cingulate	19	106.39	64.46	7	98.24	78.66	7	103.95	48.45	NS
Entorhinal	19	77.34	44.50	7	65.01	31.57	7	58.49	33.66	NS
Frontal	19	89.91	55.41	7	94.35	32.33	7	115.74	94.82	NS
Parietal	19	98.61	65.63	7	84.88	43.32	7	65.06	31.57	NS
Putamen	19	113.58	86.23	7	108.49	51.61	7	131.32	69.68	NS
Entorhinal	19	94.17	70.21	7	92.35	42.74	7	69.65	27.86	NS
Trigone	16	747.98	694.51	6	690.52	513.68	6	180.32	166.89	NS

Comparison of mean MAG:PLP1 ratio in groups stratified according to Braak tangle stage (BS) groups (0-II, III-IV, and V-VI), Thal phase groups (0-2, 3-4 and 5-6), Cerebral amyloid angiopathy (CAA; 0-3) and cerebral small vessel disease (SVD; 0-4) scores. CAA and SVD scores are derived on frontal lobe scores for which a complete dataset was available. N = number in each group. Mean = average per group. SD = standard deviation. P-value based on 1-WAY ANOVA.

Supplementary Table 4: Regional VEGF-A and EDN1 analysis

3A)VEGFA

**Braak stage:**		**BS 0-II**			**BS III-IV**			**BS V-VI**		
**Region**	**N**	**Mean**	**SD**	**N**	**Mean**	**SD**	**N**	**Mean**	**SD**	**p-value**
Calcarine	7	-0.84	0.33	17	0.12	0.50	10	-0.17	0.51	p = 0.0004
Cingulate	7	-0.59	0.35	17	0.67	0.87	11	1.35	1.40	p = 0.0016
Entorhinal	8	-0.78	0.39	17	0.43	0.74	10	0.34	0.86	p = 0.0011
Frontal	8	-0.71	0.32	17	0.56	0.95	11	1.27	1.37	p = 0.0008
Parietal	7	-0.76	0.35	16	0.45	0.88	11	-0.06	0.81	p = 0.0062
Putamen	7	-1.01	0.15	17	-0.60	0.33	11	-0.52	0.76	NS
Entorhinal	8	-0.74	0.53	17	0.43	0.65	11	0.81	0.94	p = 0.0002
Trigone	4	-1.18	0.14	17	-0.92	0.22	7	-0.99	0.37	NS

Thal Phase:		*Thal 0-2*			*Thal 3-4*			*Thal 5-6*		
Region	*N*	*Mean*	*SD*	*N*	*Mean*	*SD*	*N*	*Mean*	*SD*	*p-value*
Calcarine	7	-0.61	0.47	15	0.07	0.62	12	-0.19	0.50	0.03
Cingulate	8	0.75	1.61	15	0.59	1.10	12	0.59	1.10	NS
Entorhinal	8	-0.09	0.97	15	0.18	0.90	12	0.20	0.77	NS
Frontal	9	0.57	1.49	15	0.41	1.07	12	0.55	1.29	NS
Parietal	8	-0.49	0.36	15	0.33	0.92	12	0.04	0.98	NS
Putamen	8	-0.53	0.67	15	-0.57	0.52	12	-0.85	0.34	NS
Entorhinal	9	0.18	1.05	15	0.29	0.85	12	0.35	0.97	NS
Trigone	5	-0.82	0.44	14	-0.96	0.21	9	-1.09	0.19	NS

CAA score:		*CAA 0*			*CAA 1*			*CAA 2-3*		
Region	*N*	*Mean*	*SD*	*N*	*Mean*	*SD*	*N*	*Mean*	*SD*	*p-value*
Calcarine	13	-0.03	0.74	8	-0.32	0.33	12	-0.31	0.41	NS
Cingulate	14	0.37	1.08	7	0.60	1.25	12	0.65	1.20	NS
Entorhinal	14	0.15	0.97	8	0.31	0.93	12	-0.16	0.52	NS
Frontal	14	0.16	1.18	8	0.73	1.48	12	0.42	0.89	NS
Parietal	13	0.32	1.10	7	-0.11	0.65	12	-0.17	0.80	NS
Putamen	13	-0.69	0.40	8	-0.76	0.20	12	-0.62	0.71	NS
Entorhinal	14	0.21	0.87	8	0.32	1.08	12	0.21	0.94	NS
Trigone	12	-0.98	0.25	6	-1.00	0.19	9	-0.97	0.35	NS

SVD score:		*SVD 0*			*SVD 1*			*SVD 2-3*		
Region	*N*	*Mean*	*SD*	*N*	*Mean*	*SD*	*N*	*Mean*	*SD*	*p-value*
Calcarine	18	-0.25	0.54	7	-0.16	0.52	7	0.09	0.79	NS
Cingulate	18	0.43	1.14	7	-0.03	0.45	7	1.32	1.34	NS
Entorhinal	19	0.06	0.86	7	-0.32	0.52	7	0.49	0.91	NS
Frontal	19	0.34	1.21	7	-0.22	0.45	7	0.83	1.15	NS
Parietal	19	0.02	0.87	6	-0.22	0.47	6	0.52	1.32	NS
Putamen	19	-0.77	0.40	6	-0.89	0.29	7	-0.28	0.71	0.04
Entorhinal	19	0.25	0.96	7	-0.24	0.42	7	0.46	0.98	NS
Trigone	15	-1.04	0.21	6	-1.03	0.20	5	-0.72	0.39	NS (0.0582)

3B) EDN1

Braak stage:		*BS 0-II*			*BS III-IV*			*BS V-VI*		
Region	*N*	*Mean*	*SD*	*N*	*Mean*	*SD*	*N*	*Mean*	*SD*	*p-value*
Calcarine	8	-0.73	0.48	17	0.60	0.94	11	0.42	1.35	p = 0.0138
Cingulate	7	-0.80	0.67	17	0.30	0.77	11	0.29	0.87	p = 0.0094
Entorhinal	8	-0.83	0.57	17	0.26	0.82	9	0.53	0.99	p = 0.0037
Frontal	8	-0.82	0.62	17	0.18	0.69	10	-0.01	0.86	p = 0.0111

Parietal	8	-0.70	0.54	17	0.35	0.82	11	0.14	1.68	NS
Putamen	8	-1.15	0.41	17	0.59	0.85	11	0.20	0.84	p < 0.0001
Entorhinal	8	-0.82	0.74	17	0.39	0.74	11	0.53	1.35	p = 0.0093
Trigone	7	-0.85	0.80	17	-0.64	0.41	10	-0.45	1.02	NS

Thal Phase:		*Thal 0-2*			*Thal 3-4*			*Thal 5-6*		
Region	*N*	*Mean*	*SD*	*N*	*Mean*	*SD*	*N*	*Mean*	*SD*	*p-value*
Calcarine	9	0.04	0.96	15	0.60	1.27	12	-0.04	0.98	NS
Cingulate	8	-0.43	0.83	15	0.25	0.86	12	0.19	0.88	NS
Entorhinal	7	-0.09	1.21	15	0.11	0.79	12	0.13	1.05	NS
Frontal	8	-0.57	0.72	15	0.18	0.81	12	-0.15	0.78	NS
Parietal	9	-0.33	0.98	15	0.33	0.97	12	-0.01	1.46	NS
Putamen	9	-0.30	1.17	15	0.27	0.99	12	0.13	0.94	NS
Entorhinal	9	-0.23	1.18	15	0.26	0.86	12	0.33	1.26	NS
Trigone	7	-0.81	0.86	15	-0.51	0.52	12	-0.66	0.84	NS

CAA score:		*CAA 0*			*CAA 1*			*CAA 2-3*		
Region	*N*	*Mean*	*SD*	*N*	*Mean*	*SD*	*N*	*Mean*	*SD =*	*p-value*
Calcarine	14	-0.09	1.02	8	0.42	0.98	12	0.41	1.23	NS
Cingulate	14	-0.24	0.86	7	0.50	0.50	11	0.15	1.03	NS
Entorhinal	14	-0.39	0.66	8	0.55	0.83	12	0.15	1.05	p = 0.0479
Frontal	14	-0.40	0.74	8	0.25	0.46	12	0.05	1.00	NS
Parietal	14	-0.24	0.87	8	0.19	0.68	12	0.22	1.58	NS
Putamen	14	0.09	1.13	8	0.18	1.06	12	-0.06	0.95	NS
Entorhinal	14	-0.38	0.82	8	0.58	0.55	12	0.39	1.28	NS (p = 0.0538)
Trigone	14	-0.75	0.65	6	-0.53	0.36	12	-0.64	0.83	NS

SVD score:		*SVD 0*			*SVD 1*			*SVD 2-3*		
Region	*N*	*Mean*	*SD*	*N*	*Mean*	*SD*	*N*	*Mean*	*SD*	*p-value*
Calcarine	19	0.12	1.09	7	0.43	1.25	7	0.26	1.14	NS
Cingulate	18	0.11	1.08	7	0.20	0.66	7	-0.12	0.60	NS
Entorhinal	19	0.01	1.03	7	0.32	0.81	6	-0.39	0.53	NS
Frontal	19	-0.08	0.98	7	0.01	0.64	7	-0.23	0.58	NS
Parietal	19	0.01	1.33	7	0.06	0.69	7	0.11	1.07	NS
Putamen	19	-0.19	1.07	7	1.00	0.66	7	-0.08	0.69	p = 0.0221
Entorhinal	19	0.17	1.25	7	0.21	0.73	7	-0.14	0.75	NS
Trigone	18	-0.61	0.76	7	-0.58	0.62	7	-0.89	0.46	NS

Comparison of average A) VEGF-A Z-scores and B) endothelin-1 (EDN1) Z-scores in groups stratified according to Braak tangle stage (BS) groups (0-II, III-IV, and V-VI), Thal phase groups (0-2, 3-4 and 5-6), Cerebral amyloid angiopathy (CAA; 0-3) and cerebral small vessel disease (SVD; 0-4) scores. CAA and SVD scores are derived on frontal lobe scores for which a complete dataset was available. N = number in each group. Mean = average per group. SD = standard deviation. P-value based on 1-WAY ANOVA.

Supplementary Table 5: Regional CD31 analysis

**Braak stage:**		**BS 0-II**			**BS III-IV**			**BS V-VI**		
**Region**	**N**	**Mean**	**SD**	**N**	**Mean**	**SD**	**N**	**Mean**	**SD**	**p-value**
Calcarine	8	-0.78	0.62	17	0.14	0.42	11	-0.42	0.54	p = 0.0004
Cingulate	8	-0.15	0.75	17	0.31	0.65	11	0.65	0.83	NS (p = 0.07)
Entorhinal	8	0.41	1.47	17	0.88	0.57	10	1.36	1.42	NS
Frontal	8	-0.17	1.05	17	0.43	0.52	11	0.82	1.33	NS
Parietal	8	-0.61	0.85	17	0.17	0.64	11	-0.30	0.78	p = 0.0460
Putamen	8	-0.65	0.99	17	-0.12	0.51	11	-0.46	0.87	NS
Entorhinal	8	-0.43	0.81	17	0.44	0.73	11	0.54	0.90	p = 0.0270
Trigone	7	-1.34	0.45	17	-1.21	0.37	11	-1.08	0.59	NS
Thal Phase:		*Thal 0-2*			*Thal 3-4*			*Thal 5-6*		
Region	*N*	*Mean*	*SD*	*N*	*Mean*	*SD*	*N*	*Mean*	*SD*	*p-value*
Calcarine	9	-0.27	0.71	15	-0.18	0.64	12	-0.28	0.59	NS
Cingulate	9	0.57	0.64	15	0.18	0.79	12	0.29	0.84	NS
Entorhinal	8	1.08	1.26	15	0.51	0.71	12	1.30	1.34	NS
Frontal	9	0.64	0.74	15	0.20	0.86	12	0.50	1.29	NS
Parietal	9	-0.01	0.58	15	-0.14	0.82	12	-0.26	0.90	NS
Putamen	9	-0.04	0.93	15	-0.38	0.67	12	-0.52	0.74	NS
Entorhinal	9	0.16	0.78	15	0.20	0.64	12	0.45	1.18	NS
Trigone	8	-0.81	0.64	15	-1.29	0.37	12	-1.33	0.28	p = 0.00260
CAA score:		*CAA 0*			*CAA 1*			*CAA 2-3*		
Region	*N*	*Mean*	*SD*	*N*	*Mean*	*SD*	*N*	*Mean*	*SD*	*p-value*
Calcarine	14	-0.25	0.55	8	0.24	0.64	12	-0.48	0.49	p = 0.0258
Cingulate	14	0.17	0.65	8	0.74	0.71	12	0.08	0.86	NS
Entorhinal	14	0.91	1.03	8	1.10	1.72	12	0.81	0.82	NS
Frontal	14	0.28	0.75	8	0.73	1.47	12	0.32	0.97	NS
Parietal	14	-0.17	0.64	8	0.22	0.75	12	-0.38	0.95	NS
Putamen	14	-0.18	0.82	8	-0.09	0.77	12	-0.72	0.67	NS
Entorhinal	14	0.07	0.80	8	0.38	0.81	12	0.44	1.07	NS
Trigone	14	-1.13	0.47	7	-1.21	0.43	12	-1.28	0.52	NS
SVD score:		*SVD 0*			*SVD 1*			*SVD 2-3*		
Region	*N*	*Mean*	*SD*	*N*	*Mean*	*SD*	*N*	*Mean*	*SD*	*p-value*
Calcarine	19	-0.36	0.62	7	0.07	0.53	7	-0.14	0.62	NS
Cingulate	19	0.15	0.85	7	0.16	0.72	7	0.57	0.47	NS
Entorhinal	19	0.95	1.46	7	0.93	0.37	7	0.73	0.64	NS
Frontal	19	0.48	1.24	7	0.27	0.67	7	0.14	0.48	NS
Parietal	19	-0.10	0.80	7	-0.17	0.99	7	-0.22	0.74	NS
Putamen	19	-0.63	0.63	7	-0.07	1.06	7	-0.06	0.60	NS
Entorhinal	19	0.32	1.01	7	0.32	0.86	7	-0.03	0.61	NS
Trigone	18	-1.32	0.35	7	-1.32	0.21	7	-0.93	0.71	NS

Comparison of average CD31 Z-scores in groups stratified according to Braak tangle stage (BS) groups (0-II, III-IV, and V-VI), Thal phase groups (0-2, 3-4 and 5-6), Cerebral amyloid angiopathy (CAA; 0-3) and cerebral small vessel disease (SVD; 0-4) scores. CAA and SVD scores are derived on frontal lobe scores for which a complete dataset was available. N = number in each group. Mean = average per group. SD = standard deviation. P-value based on 1-WAY ANOVA.

Supplementary Table 6: Regional PDGFRβ:CD31 analysis

**Braak stage:**		**BS 0-II**			**BS III-IV**			**BS V-VI**		
**Region**	**N**	**Mean**	**SD**	**N**	**Mean**	**SD**	**N**	**Mean**	**SD**	**p-value**
Calcarine	8	1.55	0.81	17	0.75	0.26	11	0.88	0.75	$p = 0.0110$
Cingulate	8	1.36	0.71	17	0.80	0.23	11	0.85	0.42	$p = 0.0135$
Entorhinal	8	1.07	0.50	17	0.62	0.26	10	0.54	0.35	$p = 0.0071$
Frontal	8	2.07	0.95	17	0.90	0.17	11	0.83	0.44	$P < 0.0001$
Parietal	8	2.10	1.46	17	0.82	0.20	11	0.91	0.58	$p = 0.0011$
Putamen	8	1.15	0.59	17	0.63	0.28	11	0.76	0.27	$p = 0.0084$
Entorhinal	8	2.07	1.17	17	0.69	0.22	11	0.77	0.46	$p < 0.0001$
Trigone	7	2.58	1.28	17	1.56	0.69	11	1.35	0.63	$p = 0.0102$

Thal Phase:		*Thal 0-2*			*Thal 3-4*			*Thal 5-6*		
Region	*N*	*Mean*	*SD*	*N*	*Mean*	*SD*	*N*	*Mean*	*SD*	*p-value*
Calcarine	9	1.46	0.89	15	0.81	0.31	12	0.80	0.63	$p = 0.0281$
Cingulate	9	1.08	0.58	15	0.91	0.40	12	0.89	0.51	NS
Entorhinal	8	0.97	0.50	15	0.67	0.31	12	0.55	0.36	NS ($p = 0.0616$)
Frontal	9	1.25	0.40	15	1.12	0.64	12	1.06	0.98	NS
Parietal	9	1.39	0.79	15	0.96	0.46	12	1.16	1.33	NS
Putamen	9	0.81	0.26	15	0.73	0.50	12	0.82	0.41	NS
Entorhinal	9	1.70	0.94	15	0.78	0.28	12	0.81	0.96	$p = 0.0127$
Trigone	8	1.73	1.18	15	1.68	0.78	12	1.71	0.96	NS

CAA score:		*CAA 0*			*CAA 1*			*CAA 2-3*		
Region	*N*	*Mean*	*SD*	*N*	*Mean*	*SD*	*N*	*Mean*	*SD*	*p-value*
Calcarine	14	0.97	0.69	8	0.94	0.29	12	0.86	0.67	NS
Cingulate	14	1.00	0.57	8	0.78	0.16	12	0.97	0.54	NS
Entorhinal	14	0.74	0.45	8	0.66	0.30	12	0.66	0.43	NS
Frontal	14	1.15	0.61	8	1.08	0.43	12	1.11	1.01	NS
Parietal	14	1.11	0.58	8	1.02	0.62	12	1.28	1.38	NS
Putamen	14	0.74	0.28	8	0.71	0.27	12	0.87	0.62	NS
Entorhinal	14	1.03	0.67	8	1.11	1.00	12	0.92	0.98	NS
Trigone	14	1.66	0.82	7	1.71	0.77	12	1.84	1.15	NS

SVD score:		*SVD 0*			*SVD 1*			*SVD 2-3*		
Region	*N*	*Mean*	*SD*	*N*	*Mean*	*SD*	*N*	*Mean*	*SD*	*p-value*
Calcarine	19	0.91	0.62	7	0.78	0.60	7	1.07	0.63	NS
Cingulate	19	0.92	0.47	7	1.04	0.58	7	0.93	0.53	NS
Entorhinal	19	0.70	0.45	7	0.67	0.35	7	0.73	0.38	NS
Frontal	19	1.11	0.60	7	0.93	0.44	7	1.36	1.22	NS
Parietal	19	1.03	0.67	7	1.00	0.46	7	1.55	1.70	NS
Putamen	19	0.83	0.43	7	0.66	0.38	7	0.76	0.52	NS
Entorhinal	19	1.00	0.85	7	0.76	0.55	7	1.31	1.12	NS
Trigone	18	1.64	0.78	7	2.01	0.74	7	1.84	1.43	NS

Comparison of the average PGDFRβ:CD31 ratio in groups stratified according to Braak tangle stage (BS) groups (0-II, III-IV, and V-VI), Thal phase groups (0-2, 3-4 and 5-6), Cerebral amyloid angiopathy (CAA; 0-3) and cerebral small vessel disease (SVD; 0-4) scores. CAA and SVD scores are derived on frontal lobe scores for which a complete dataset was available. N = number in each group. Mean = average per group. SD = standard deviation. P-value based on 1-WAY ANOVA.

Supplementary Table 7: Regional Fibrinogen analysis

**Braak stage:**		**BS 0-II**			**BS III-IV**			**BS V-VI**		
**Region**	**N**	**Mean**	**SD**	**N**	**Mean**	**SD**	**N**	**Mean**	**SD**	**p-value**
Calcarine	8	-0.35	0.52	17	-0.19	0.51	11	1.07	0.97	p < 0.0001
Cingulate	8	-0.44	0.60	17	-0.31	0.56	11	0.95	1.36	p = 0.0013
Entorhinal	8	-0.35	0.52	17	-0.19	0.51	10	1.07	0.97	p = 0.0007
Frontal	8	-0.35	0.52	16	-0.19	0.51	11	1.07	0.97	p = 0.0081
Parietal	8	-0.44	0.59	17	-0.52	0.42	11	0.76	1.24	p = 0.0005
Putamen	8	0.06	0.68	17	0.16	0.73	11	1.13	0.92	p = 0.0046
Entorhinal	8	-0.27	0.82	17	-0.50	0.38	11	1.11	0.92	p < 0.0001
Trigone	7	-0.82	0.54	17	-0.87	0.30	11	0.20	1.01	p = 0.0004
Thal stage:		*Thal 0-2*			*Thal 3-4*			*Thal 5-6*		
Region	*N*	*Mean*	*SD*	*N*	*Mean*	*SD*	*N*	*Mean*	*SD*	*p-value*
Calcarine	9	0.53	1.00	15	-0.19	0.62	12	0.32	1.05	NS
Cingulate	9	0.28	1.00	15	-0.32	0.65	12	0.33	1.40	NS
Entorhinal	8	-0.03	1.01	15	-0.28	0.64	12	0.44	1.70	NS
Frontal	9	0.21	1.19	15	-0.34	0.69	11	0.02	1.18	NS
Parietal	9	-0.08	0.65	15	-0.28	0.84	12	0.08	1.30	NS
Putamen	9	0.70	0.96	15	0.20	0.81	12	0.52	0.94	NS
Entorhinal	9	0.27	0.93	15	-0.32	0.66	12	0.32	1.26	NS
Trigone	8	-0.04	0.95	15	-0.78	0.84	12	-0.52	0.51	NS
CAA score:		*CAA 0*			*CAA 1*			*CAA 2-3*		
Region	*N*	*Mean*	*SD*	*N*	*Mean*	*SD*	*N*	*Mean*	*SD*	*p-value*
Calcarine	14	-0.23	0.46	8	0.21	0.82	12	0.28	1.04	NS
Cingulate	14	-0.29	0.67	8	0.41	1.61	12	-0.05	0.83	NS
Entorhinal	14	-0.39	0.61	8	0.58	1.99	12	0.07	0.95	NS
Frontal	13	-0.38	0.75	8	0.23	1.41	12	-0.08	0.97	NS
Parietal	14	-0.49	0.44	8	0.24	1.35	12	-0.03	1.08	NS
Putamen	14	0.16	0.74	8	0.62	1.16	12	0.45	0.84	NS
Entorhinal	14	-0.38	0.69	8	0.00	0.95	12	0.38	1.16	NS
Trigone	14	-0.74	0.46	7	-0.56	0.66	11	-0.72	0.57	NS
SVD score:		*SVD 0*			*SVD 1*			*SVD 2-3*		
Region	*N*	*Mean*	*SD*	*N*	*Mean*	*SD*	*N*	*Mean*	*SD*	*p-value*
Calcarine	19	0.07	0.99	8	-0.22	0.32	7	0.15	0.51	NS
Cingulate	19	-0.04	1.23	8	-0.26	0.65	7	0.04	0.73	NS
Entorhinal	19	-0.01	1.46	8	-0.24	0.61	7	-0.01	0.67	NS
Frontal	18	-0.23	1.01	8	-0.34	0.71	7	-0.04	0.79	NS
Parietal	19	-0.10	1.12	8	-0.59	0.35	7	0.03	0.98	NS
Putamen	19	0.39	0.88	8	0.22	0.46	7	0.18	0.93	NS
Entorhinal	19	0.02	1.10	8	-0.13	0.96	7	-0.19	0.61	NS
Trigone	18	-0.73	0.48	7	-0.65	0.62	7	-0.48	1.15	NS

Comparison of average Fibrinogen Z-scores in groups stratified according to Braak tangle stage (BS) groups (0-II, III-IV, and V-VI), Thal phase groups (0-2, 3-4 and 5-6), Cerebral amyloid angiopathy (CAA; 0-3) and cerebral small vessel disease (SVD; 0-4) scores. CAA and SVD scores are derived on frontal lobe scores for which a complete dataset was available. N = number in each group. Mean = average per group. SD = standard deviation. P-value based on 1-WAY ANOVA.

Supplementary Table 8. Relationship between VEGF-A, EDN1 and CD31 and the MAG:PLP1 ratio across brain regions

**8A) Entire cohort**				**8B) BS 0-II**			**BS III-IV**			**BS V-VI**		
MAG/PLP:	*VEGF*	*EDN1*	*CD31*	*VEGF*	*EDN1*	*CD31*	*VEGF*	*EDN1*	*CD31*	*VEGF*	EDN1	CD31
Calcarine	-0.53**	-0.38*	-0.46**	NS	NS	NS	NS	NS	-0.65**	NS	NS	NS
Cingulate	-0.40*	-0.43*	NS	NS	NS	NS	NS	NS	0.50*	NS	NS	NS
Entorhinal	-0.67***	-0.43*	NS	NS	NS	NS	NS	NS	NS	NS	NS	NS
Frontal	-0.46**	-0.40*	-0.37*	NS	NS	NS	NS	NS	NS	NS	NS	-0.734*
Parietal	-0.52**	NS	-0.33*	NS	NS	NS	-0.54*	NS	NS	NS	NS	NS
Putamen	NS	-0.42**	NS	NS	NS	NS	NS	NS	-0.68**	NS	NS	NS
Temporal	-0.68***	0.35*	-0.62***	NS	NS	NS	-0.49*	0.48*	NS	-0.614*	NS	-0.758**
Trigone	-0.68***	NS	-0.53**	NS	NS	NS	-0.69**	NS	-0.63*	NS	NS	NS

Pearson's correlation coefficients (r) were calculated to assess the relationship between the MAG:PLP1 ratio and concentrations of VEGF-A, endothelin-1 (EDN1), and CD31, all measured by ELISA, in A) the entire cohort and B) individual Braak tangle stage groups (0-II, III-IV, V-VI), in the different brain regions. * $P > 0.05$, ** $P < 0.01$, *** $P < 0.001$. NS = non-significant.

Supplementary Table 9. Relationship between VEGF-A, EDN1 And CD31

Region	VEGF/CD31	CD31/EDN1	VEGF/EDN1
Calcarine	0.40*	.51**	.40*
Cingulate	0.70***	0.42*	NS
Entorhinal	0.32 (0.06)	NS	0.41*
Frontal	0.59***	NS	NS
Parietal	0.43*	NS	NS
Putamen	0.44*	NS	NS
Temporal	0.52**	NS	NS
Trigone	0.67***	NS	NS

Pearson's correlation coefficients (r) were calculated to assess the relationship between VEGF-A and CD31, endothelin-1 (EDN1) and CD31, and VEGF-A and CD31, across the entire cohort, in the different brain regions. * $P > 0.05$, ** $P < 0.01$, *** $P < 0.001$. NS = non-significant.

Supplementary Figure 1. Regional Aβ and tau concentrations

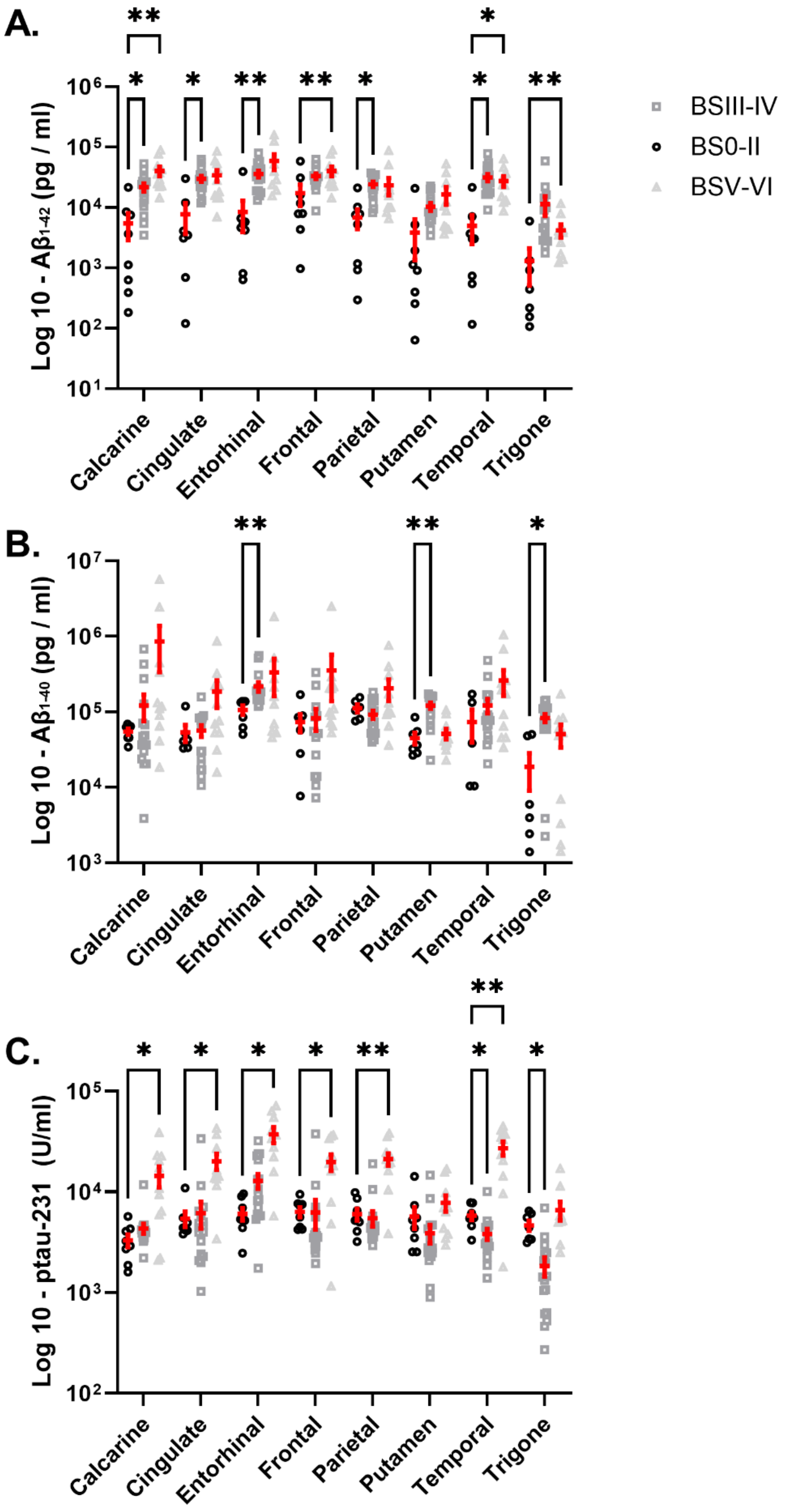

Concentrations of (A) $A\beta_{1-42}$, (B) $A\beta_{1-40}$ and (C) ptau-231 measured by ELISA in the different brain regions assessed in this study. We used two-way mixed-effects ANOVA (accounting for regions with missing data) with Dunnet's multiple comparisons test, to compare levels between Braak tangle stage (BS) groups in the different brain regions. BS0-II was used as the comparator group. Each dot represents the average of a duplicate measurement in a single brain region. Mean ± SEM are shown. * $p < 0.05$ ** $p < 0.01$.

Supplementary Table 10. Correlations between pathological markers across brain regions

**Region:**	**Aβ_{1-42}/Aβ_{1-40}**	**Aβ_{1-42}/ptau231**	**Aβ_{1-40}/ptau-231**
Calcarine	0.55***	0.48**	0.50*
Cingulate	0.58***	0.53***	0.53***
Entorhinal	0.72***	0.64***	0.53***
Frontal	0.44*	NS	0.46**
Parietal	0.70***	0.35*	0.60***
Putamen	NS	NS	NS
Temporal	0.70***	NS	0.31 (0.076)
Trigone	NS	NS	NS

Pearson's correlation coefficients (r) were calculated to assess the relationship between Aβ_{1-42} and Aβ_{1-40}, Aβ_{1-42} and ptau231, Aβ_{1-40} and ptau231 across the entire cohort, in the different brain regions. * $P > 0.05$, ** $P < 0.01$, *** $P < 0.001$. NS = non-significant.

Supplementary Table 11. Relationship between vascular and pathological markers across brain regions

	**Cohort:**			**BS 0-II:**			**BS III-IV:**			**BS V-VI:**		
MAG/PLP1:	***$A\beta_{42}$***	***$A\beta_{40}$***	***ptau231***	***$A\beta_{42}$***	***$A\beta_{40}$***	***ptau231***	***$A\beta_{42}$***	***$A\beta_{40}$***	***ptau231***	***$A\beta_{42}$***	***$A\beta_{40}$***	***ptau231***
Calcarine	NS	NS	NS	-0.82*	NS	NS	NS	NS	NS	NS	NS	NS
Cingulate	-0.41*	NS	NS	NS	NS	NS	NS	NS	NS	NS	NS	NS
Entorhinal	NS	NS	NS	NS	NS	NS	NS	NS	NS	NS	NS	NS
Frontal	-0.32 (0.057)	NS	NS	NS	NS	NS	NS	NS	NS	NS	NS	NS
Parietal	NS	NS	NS	NS	NS	0.85**	NS	NS	NS	NS	NS	NS
Putamen	-0.317 (0.068)	NS	NS	NS	0.89**	NS	NS	0.67**	NS	NS	NS	NS
Temporal	NS	NS	NS	NS	NS	NS	NS	NS	NS	NS	NS	NS
Trigone	NS	-0.61***	NS	NS	NS	NS	NS	-0.57*	0.78**	NS	-0.68*	NS
VEGF-A:	*$A\beta_{42}$*	*$A\beta_{40}$*	*Tau*	*$A\beta_{42}$*	*$A\beta_{40}$*	*ptau231*	*$A\beta_{42}$*	*$A\beta_{40}$*	*Tau-231*	*$A\beta_{42}$*	*$A\beta_{40}$*	*ptau231*
Calcarine	NS	NS	NS	NS	NS	NS	NS	NS	NS	NS	NS	NS
Cingulate	NS	NS	NS	NS	NS	NS	NS	-0.54*	NS	-0.63*	NS	NS
Entorhinal	NS	NS	NS	NS	NS	NS	NS	NS	NS	NS	NS	NS
Frontal	NS	NS	NS	NS	NS	NS	NS	NS	NS	NS	NS	NS
Parietal	NS	NS	NS	NS	NS	NS	NS	NS	NS	NS	NS	NS
Putamen	NS	NS	NS	NS	0.87*	NS	-0.49 (0.05)	NS	0.45 (0.06)	-0.58 (0.08)	NS	NS
Temporal	NS	NS	NS	NS	NS	NS	NS	NS	NS	NS	NS	NS
Trigone	NS	0.53**	NS	NS	NS	NS	NS	NS	-0.57*	NS	NS	NS
EDN1:	*$A\beta_{42}$*	*$A\beta_{40}$*	*Tau*	*$A\beta_{42}$*	*$A\beta_{40}$*	*ptau231*	*Ab42*	*$A\beta_{40}$*	*Tau-231*	*$A\beta_{42}$*	*$A\beta_{40}$*	*ptau231*
Calcarine	0.39*	NS	0.36*	NS	NS	NS	0.45 (0.07)	NS	NS	NS	NS	NS
Cingulate	0.43*	NS	0.32 (0.052)	0.96**	NS	NS	NS	NS	0.55*	NS	NS	NS
Entorhinal	NS	NS	NS	0.93**	NS	NS	NS	0.42 (0.09)	0.44 (0.08)	NS	NS	NS
Frontal	NS	NS	NS	0.72*	NS	NS	NS	NS	0.61**	NS	NS	NS
Parietal	NS	NS	NS	0.88**	NS	NS	NS	NS	NS	NS	NS	NS
Putamen	NS	NS	NS	NS	NS	NS	NS	NS	NS	NS	NS	NS
Temporal	NS	NS	NS	0.84***	NS	NS	NS	NS	0.53*	NS	NS	NS
Trigone	NS	NS	0.46**	NS	NS	NS	NS	-0.48 (0.05)	NS	NS	NS	0.673*
CD31:	*$A\beta_{42}$*	*$A\beta_{40}$*	*Tau*	*$A\beta_{42}$*	*$A\beta_{40}$*	*ptau231*	*Ab42*	*$A\beta_{40}$*	*Tau-231*	*$A\beta_{42}$*	*$A\beta_{40}$*	*ptau231*
Calcarine	NS	NS	NS	NS	NS	NS	NS	NS	NS	NS	NS	NS
Cingulate	NS	NS	NS	NS	NS	-0.80*	NS	-0.56*	-0.54*	NS	NS	NS
Entorhinal	NS	NS	NS	NS	NS	-0.74*	NS	NS	0.64**	NS	NS	NS
Frontal	NS	NS	NS	-0.659 (0.076)	NS	NS	NS	NS	0.62**	NS	NS	NS
Parietal	0.34*	NS	NS	NS	-0.89*	NS	NS	NS	NS	NS	NS	NS
Putamen	-0.39*	-0.42*	-0.45**	NS	NS	NS	NS	0.66**	NS	-0.65*	NS	-0.71*
Temporal	NS	NS	NS	NS	-0.84 (0.08)	NS	NS	NS	NS	NS	NS	NS
Trigone	NS	NS	NS	NS	0.85*	NS	NS	NS	NS	NS	NS	NS

Pearson's correlation coefficients (r) were calculated to assess the relationships between the MAG:PLP1 ratio, and concentrations of VEGF-A, endothelin-1 (EDN1) and CD31, with AD neuropathological markers $A\beta_{1-40}$, $A\beta_{1-42}$ and ptau231 levels, in the different brain regions. Correlation

analysis was performed across the entire cohort and also in individual groups stratified by Braak tangle stage. * $P > 0.05$, ** $P < 0.01$, *** $P < 0.001$. NS = non-significant.

Supplementary Table 12. Relative level of angiogenic proteins in relation to Braak tangle stage groups across brain regions

	BS0-II (n = 3)		BSV-VI (n = 3)		BSV-VI (n = 4)		2-WAY ANOVA (Dunnet's)	
							BS0-II vs BSIII-IV	BS0-II vs BSV-VI
	Average	SD	Average	SD	Average	SD		
Activin A	72.35	22.95	69.54	16.73	97.92	19.19	NS	NS
ADAMTS	80.77	65.08	69.39	26.10	82.49	21.63	NS	NS
Angiogenin	386.64	163.42	587.28	169.83	289.39	156.19	NS	NS
Angiopoietin-1	77.76	51.12	110.22	77.56	65.99	24.38	NS	NS
Angiopoietin-2	119.54	22.37	161.11	42.96	142.18	45.53	NS	NS
Angiostatin /Plasminogen	71.52	19.70	109.88	82.27	73.71	27.00	NS	NS
Amphiregulin	100.99	22.15	169.77	155.39	109.11	16.19	NS	NS
Artemin	215.55	54.77	257.04	124.01	143.69	39.96	NS	NS
Coagulation Factor III	2477.69	275.88	2021.45	493.90	2672.04	584.01	NS	NS
CXCL16	273.09	108.15	235.60	84.34	252.27	188.64	NS	NS
DPPIV/CD26	210.92	130.29	285.80	166.90	240.32	174.21	NS	NS
EGF	130.44	45.00	141.38	106.63	130.17	44.00	NS	NS
EG-VEGF	152.02	37.52	183.34	73.85	150.26	28.21	NS	NS
Endoglin	205.02	51.88	457.06	66.45	227.73	147.39	NS	NS
Endostatin /CollagenCVIII	222.23	126.17	384.71	395.24	376.92	209.23	NS	NS
Endothelin-1	234.95	96.07	293.26	134.64	277.56	230.63	NS	NS
FGF acidic	2096.64	330.03	2384.02	440.32	2593.53	327.05	NS	***
FGF basic	763.42	137.41	1424.63	336.06	1052.29	492.99	****	NS
FGF-4	135.53	28.89	117.89	106.43	128.81	51.66	NS	NS
FGF-7	142.16	30.82	65.98	28.40	95.84	25.20	NS	NS
GDNF	168.03	45.27	114.09	15.39	139.51	49.60	NS	NS
GM-CSF	107.51	47.26	62.64	18.11	80.55	21.81	NS	NS
HB-EGF	189.47	70.08	260.46	30.74	219.35	70.32	NS	NS
HGF	210.39	124.13	274.86	243.68	283.13	186.02	NS	NS
IGFBP-1	714.31	89.84	485.81	73.96	438.29	88.75	NS (0.1)	NS (0.1)
IGFBP-2	679.14	246.06	631.58	160.61	758.99	337.75	NS	NS
IGFBP-3	89.18	15.28	125.90	24.54	138.11	95.33	NS	NS
IL-1B	56.64	40.35	106.62	50.30	80.10	34.60	NS	NS
IL-8	73.89	22.56	114.44	36.82	100.75	33.69	NS	NS
LAP	74.18	32.67	77.90	13.01	110.20	34.65	NS	NS
Leptin	65.77	33.02	103.30	61.82	134.61	40.97	NS	NS
MCP	63.45	38.77	99.95	83.43	89.48	31.99	NS	NS
MIP-ialpha	117.23	37.18	77.33	36.49	108.45	36.26	NS	NS
MMP-8	426.50	152.95	745.19	340.38	1300.62	711.89	*	****
MMP-9	825.15	415.82	1260.14	729.53	1359.50	462.87	**	***
NRG1-B1	76.09	20.98	100.43	74.31	68.58	4.50	NS	NS
PTX3	138.01	30.71	180.17	50.96	121.11	40.08	NS	NS

PD-ECGF	125.24	63.42	176.95	49.71	131.96	58.82	NS	NS
PDGF-AA	140.98	27.95	263.13	243.83	113.03	29.58	NS	NS
PDGF-AB/PDGF-BB	84.53	37.54	114.27	65.20	71.46	22.26	NS	NS
Persephin	135.72	14.39	142.26	59.72	120.54	36.68	NS	NS
PF4	348.88	223.41	762.83	468.90	857.01	305.79	**	***
PlGF	171.28	75.05	202.31	96.58	231.41	51.62	NS	NS
Prolactin	52.94	35.68	112.30	58.48	106.22	29.00	NS	NS
Serpin B5	70.63	20.06	83.39	25.29	109.16	24.09	NS	NS
Serpin E	247.96	63.41	306.77	75.52	367.59	71.26	NS	NS
Serpin F	3391.61	272.13	3217.35	333.50	3267.69	215.40	NS	NS
TIMP-1	199.56	70.62	407.28	88.74	233.30	44.90	NS	NS
TIMP-4	142.22	49.49	177.00	47.54	153.92	62.48	NS	NS
TSP-1	120.74	117.23	284.71	126.44	125.97	55.06	NS	NS
TSP-2	86.25	38.07	134.03	74.99	104.22	18.61	NS	NS
uPA	60.22	35.69	129.63	60.63	111.21	16.93	NS	NS
Vasohibin	95.60	67.74	90.37	78.46	107.90	48.47	NS	NS
VEGF-A	79.15	69.07	127.36	40.17	126.67	19.23	NS	NS
VEGF-C	69.78	31.72	85.59	45.71	99.46	30.63	NS	NS

Relative levels of 55 angiogenic protein markers in Braak tangle stage (BS) groups: BS0-II Low-pathology controls (n = 3); BSIII-IV intermediate pathology (n = 3) and BSV-VI late-stage AD pathology. The average values and standard deviations are shown for all proteins. A 2-WAY ANOVA with Dunnet's post-hoc was used to identify angiogenic proteins that varied across groups. * $P < 0.05$, ** $P < 0.01$, **** $P < 0.0001$. NS = non-significant.

Supplementary Table 13. Pearson's correlation co-efficients between PDGFRβ:CD31 ratio and markers of AD pathology ($Aβ_{1-42}$, $Aβ_{1-40}$, and ptau-231) and cerebrovascular dysfunction (MAG:PLP1 ratio, VEGF-A and EDN1).

PDGFRβ: CD31:	*$Aβ_{1-42}$*	*$Aβ_{1-40}$*	*ptau231*	*MAG: PLP1*	*VEGF-A*	*EDN1*	*FB*
Calcarine	-0.54***	NS	-0.28 (0.1)	0.34*	-0.48**	-0.30 (0.08)	NS
Cingulate	-0.43**	NS	NS	NS	-0.31 (0.07)	-0.434**	NS
Entorhinal	-0.38*	-0.30(0.1)	-0.399*	NS	-0.67***	-0.38*	NS
Frontal	-0.43**	NS	NS	0.69**	-0.36*	-0.35*	NS
Parietal	-0.40*	-0.31 (0.08)	NS	NS	NS	NS	NS
Putamen	NS	NS	NS	0.37*	NS	-0.34*	NS
Temporal	-0.52***	-0.35*	NS	0.60***	-0.36*	-0.474**	NS
Trigone	NS	NS	NS	NS	-0.34 (0.08)	NS	NS

* P < 0.05, ** P < 0.01, *** P < 0.001. NS = non-significant.

Supplementary Table 14. Pearson's correlation coefficients between fibrinogen levels and markers of AD pathology ($A\beta_{1-42}$, $A\beta_{1-40}$, and ptau-231) and markers of cerebrovascular dysfunction (MAG:PLP1 ratio, VEGF-A and EDN1).

Fibrinogen:	$A\beta_{1-42}$	$A\beta_{1-40}$	*ptau231*	*MAG: PLP1*	*VEGF-A*	*EDN1*	*CD31*	*PDGFRβ: CD31*
Calcarine	NS	NS	0.33 (0.05)	NS	NS	NS	NS	NS
Cingulate	NS	NS	0.29 (0.09)	NS	0.69***	NS	0.64***	NS
Entorhinal	NS	NS	NS	-0.38*	0.43**	0.35*	0.53**	NS
Frontal	NS	NS	NS	NS	0.67***	NS	0.56***	NS
Parietal	NS	NS	0.36*	NS	0.37*	NS	NS	NS
Putamen	NS	NS	NS	-0.29 (0.08)	NS	NS	NS	NS
Temporal	NS	NS	0.59***	NS	0.47**	NS	0.47**	NS
Trigone	NS	NS	0.39*	-0.35 (0.06)	NS	NS	NS	NS

* $P < 0.05$, ** $P < 0.01$, *** $P < 0.001$. NS = non-significant.

Supplementary Table 15. Correlation of fibrinogen levels with markers of disease pathogenesis and cerebral hypoperfusion in the different brain regions, stratified according to Braak tangle stage.

(A) BS0-II

Fibrinogen:	*Aβ$_{1-42}$*	*Aβ$_{1-40}$*	*ptau231*	*MAG: PLP1*	*VEGF-A*	*EDN1*	*CD31*	*PDGFRβ: CD31*
Calcarine	NS	0.86*	NS	NS	NS	0.65 (0.08)	0.70 (0.05)	NS
Cingulate	NS	NS	NS	NS	0.88**	NS	NS	NS
Entorhinal	NS	0.78*	NS	NS	NS	0.69 (0.09)	NS	NS
Frontal	NS	NS	NS	NS	NS	NS	0.66 (0.09)	NS
Parietal	NS	NS	NS	NS	NS	0.66 (0.08)	NS	NS
Putamen	0.76*	NS	NS	NS	NS	NS	NS	NS
Temporal	NS	NS	NS	NS	NS	0.83*	NS	NS
Trigone	NS	NS	NS	NS	NS	NS	NS	NS

(B)BSIII-IV

Fibrinogen:	*Aβ$_{1-42}$*	*Aβ$_{1-40}$*	*ptau231*	*MAG: PLP1*	*VEGF-A*	*EDN1*	*CD31*	*PDGFRβ: CD31*
Calcarine	NS	NS	NS	NS	NS	NS	NS	NS
Cingulate	NS	NS	NS	NS	NS	NS	NS	NS
Entorhinal	NS	NS	NS	NS	NS	NS	NS	NS
Frontal	NS	NS	NS	NS	NS	NS	NS	NS
Parietal	NS	NS	NS	NS	0.61*	NS	NS	NS
Putamen	NS	NS	NS	NS	NS	NS	NS	NS
Temporal	NS	NS	NS	NS	NS	NS	NS	NS
Trigone	NS	NS	NS	NS	NS	NS	NS	NS

(C) BSV-VI

Fibrinogen:	*Aβ$_{1-42}$*	*Aβ$_{1-40}$*	*ptau231*	*MAG: PLP1*	*VEGF-A*	*EDN1*	*CD31*	*PDGFRβ: CD31*
Calcarine	-0.69*	NS	NS	NS	NS	NS	NS	NS
Cingulate	NS	NS	NS	NS	0.79**	NS	0.839**	NS
Entorhinal	NS	NS	-0.67*	-0.68*	0.73*	NS	0.732*	NS
Frontal	NS	NS	NS	NS	0.73*	NS	0.613*	NS
Parietal	NS	NS	NS	-0.67*	0.76**	NS	NS	NS
Putamen	NS	NS	-0.65*	NS	NS	NS	0.760**	NS
Temporal	NS	NS	NS	-0.55*	0.61*	NS	0.758**	NS
Trigone	NS	NS	NS	-0.58 (0.05)	NS	NS	NS	NS

Supplementary Figure 2. Angiogenic proteins correlate with fibrinogen levels

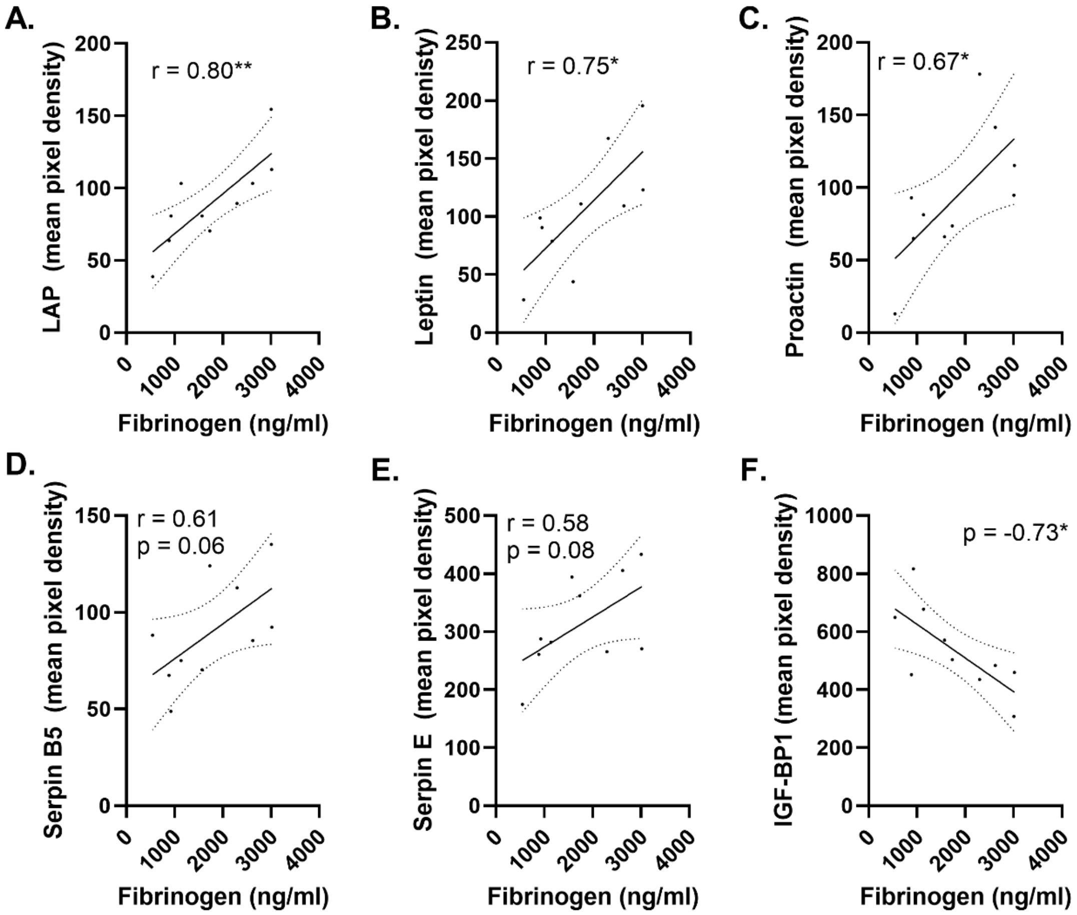

Correlations analysis between angiogenic proteins and fibrinogen level, a marker of BBB leakiness, in the temporal cortex. A-F. Linear regression and 95% confidence intervals are shown. Each dot represents the average of a duplicate measurement. Mean ± SEM are shown. * $p < 0.05$ ** $p < 0.01$.

Supplementary Table 16. List of unique UK Brain Bank identifier numbers for case used in this study.

ID:	Brain Bank:	Brain Bank UK ID no:
1	SWDBB	BBN006.30165
2	SWDBB	BBN006.36102
3	SWDBB	BBN006.36107
4	SWDBB	BBN006.36170
5	KCL	BBN002.32844
6	KCL	BBN002.28858
7	KCL	BBN_24243
8	KCL	BBN002.36130
9	SWDBB	BBN006.35560
10	SWDBB	BBN006.28710
11	SWDBB	BBN006.29640
12	SWDBB	BBN006.29894
13	SWDBB	BBN006.29917
14	SWDBB	BBN006.30034
15	SWDBB	BBN006.31445
16	SWDBB	BBN006.33677
17	SWDBB	BBN006.33699
18	SWDBB	BBN006.33731
19	SWDBB	BBN006.37600
20	SWDBB	BBN006.37559
21	SWDBB	BBN006.37415
22	SWDBB	BBN006.37118
23	SWDBB	BBN006.26344
24	SWDBB	BBN006.37663
25	SWDBB	BBN006.35417
26	SWDBB	BBN006.30889
27	SWDBB	BBN006.34151
28	SWDBB	BBN006.35453
29	SWDBB	BBN006.35513
30	KCL	BBN002.30840
31	KCL	BBN002.26278
32	KCL	BBN002.32628
33	KCL	BBN_20618
34	KCL	BBN_9984
35	KCL	BBN_9980
36	KCL	BBN002.35951
